# Nutritional adaptations to early maize cultivation: Earliest isotopic evidence of maize-based animal provisioning in the Neotropics

**DOI:** 10.1126/sciadv.aec3522

**Published:** 2026-07-08

**Authors:** Nadia C. Neff, Geraldine Busquets-Vass, Erin E. Ray, Mark Robinson, Amy E. Thompson, Jose Mes, Douglas J. Kennett, Seth D. Newsome, Keith M. Prufer

**Affiliations:** ^1^ Department of Anthropology, University of New Mexico, Albuquerque, NM, USA.; ^2^ Center for Stable Isotopes, University of New Mexico, Albuquerque, NM, USA.; ^3^ Department of Biology, University of New Mexico, Albuquerque, NM, USA.; ^4^ Centro de Investigación Científica y de Educación Superior de Ensenada, Unidad Académica La Paz, Baja California Sur, Mexico.; ^5^ Department of Archaeology, University of Exeter, Exeter, Devon, UK.; ^6^ Department of Geography and the Environment, University of Texas at Austin, Austin, TX, USA.; ^7^ Uchben’kaj Kin Ahaw Association, Santa Cruz, Toledo District, Belize.; ^8^ Department of Anthropology, University of California, Santa Barbara, Santa Barbara, CA, USA.

## Abstract

The adoption of maize as a dietary staple shaped human societies. While a reliable carbohydrate-rich source, its inherent nutritional limitations posed substantial challenges. Maize is deficient in lysine, an essential amino acid crucial for maintaining balanced health. Maize-dependent diets, therefore, necessitated complementary dietary strategies. We report amino acid stable carbon isotope data from 39 directly dated humans from southern Belize [6100 to 1100 before present (B.P.)] to investigate how early populations mitigated nutritional deficiencies. Concentration-dependent mixing model results indicate that protein supplementation from maize-eating animals contributed maize-derived lysine to human diets through trophic magnification (elevated proportions of isotopically distinct nutrients in tissues from trophic transfer). Our results indicate that such strategies were in place by 6100 B.P., consistent with evidence of early maize cultivation but predating reliance by ~2000 years. Our findings highlight early coevolutionary dynamics linking maize cultivation and human-animal provisioning relationships, deepening understandings of adaptive food systems during agricultural transitions and offering insights into nutritional strategies underpinning sustainable subsistence.

## Teaser

Carbon amino acid isotopes suggest that maize-provisioned animals were central to Neotropical human nutrition by 6100 B.P.

## INTRODUCTION

The transition toward agriculture, from the management of plants and animals to reliance on farming, catalyzed demographic expansion and socioeconomic complexity in ways that profoundly influenced human health and nutrition (*1*, *2*). Cereal grains were among the most important domesticated crops, valued for their reliability, surplus potential, and suitability as food and fodder (*3*). In the Neotropics, maize (*Zea mays* ssp. *mays*) ultimately became a dietary staple, shaping the health outcomes of early communities (*4*). Despite its global importance, diets dominated by maize present nutritional challenges due to its relatively low protein content and deficiencies in essential nutrients (*5*). These limitations likely prompted early farming societies to adopt innovative strategies to mitigate nutritional gaps, such as maize processing techniques (*6*), consuming plants with complementary nutrient profiles (*7*), or investing in animals to ensure a reliable source of protein (*8*). These strategies varied across regions and were closely linked with the coevolutionary processes of plant and animal management, resulting in multiple food production pathways (*9*). As the investment and reliance on maize increased in Mesoamerica (*10*), provisioning animals with maize likely played a crucial role in addressing potential protein deficiencies and enhancing human diet quality (*11*). Nevertheless, our understanding of how subsistence strategies shaped nutritional outcomes during the transition to agriculture remains limited. This limitation reflects the complexity of ancient food systems and the historical lack of tools for reconstructing nutrient assimilation at the macromolecular level. By examining the strategies used by early Mesoamerican communities to optimize maize-dependent diets, we offer critical insights into how precolonial societies responded to ecological and nutritional challenges, insights that remain relevant today as modern food systems struggle with similar issues of food security, sustainability, and human nutrition.

Genetic data suggest that maize domestication was underway by approximately 9000 years ago in southwest Mexico (*12*). Phylogenetic estimates further indicate divergence between maize and its wild grass progenitor, *Zea mays* ssp. *parviglumis*, perhaps around 12,000 B.P. (*13*); however, this estimate likely reflects population structure within early *Zea* lineages rather than directly reflecting the onset of domestication, which, archaeologically, is constrained to later periods (*13*). By ~7500 B.P., maize cultivation was established within Mesoamerica (*14*) and had begun to expand into parts of South America by ~6500 B.P. (*15*), while appearing much later in North America (*16*, *17*). By 4000 B.P., it was the dietary staple (*18*). In Mesoamerica, this transition coincided with increased sedentism, intensified land management, and the consequent depletion of wild resources near settlements (*11*). These changes necessitated early communities to adapt other core dietary elements to address the potential nutritional imbalances of maize-dominant diets. Maize uses the C_4_ photosynthetic pathway, making its carbon isotope (δ^13^C) value much higher [−12 to −9‰ (per mil)] and easily distinguishable from most native Neotropical plants that rely on the C_3_ pathway (−34 to −23‰) (*19*), providing an invaluable proxy for tracing the integration of maize into human and animal diets and by extension for agricultural intensification in the Neotropics (*20*). While maize’s distinct isotopic signature provides a valuable marker of agricultural intensification in this region, traditional bulk tissue stable isotope analysis lacks the resolution to disentangle the contributions of dietary macromolecules (e.g., proteins versus carbohydrates) (*21*). Advances in stable isotope analysis, particularly the ability to examine the δ^13^C values of the smaller compounds that make up bulk tissues, offer a framework for tracing the relative contributions of domestic plants and animals versus wild resources in archaeological diets. When integrated with nutritional data, these approaches can help mitigate some of the interpretive limitations of traditional isotopic analyses, providing crucial insights into the nutritional challenges and dietary trade-offs early farming communities faced (*4*).

Isotopic evidence across the Americas documents varied human-animal relationships involving maize provisioning (*22*–*24*), ranging from ceremonial and trade-related use (*25*–*27*) to household-level care (*10*, *28*, *29*). While direct evidence of animal management before 2200 B.P. in Mesoamerica is limited (*30*), shifts in human δ^13^C values after 4600 B.P. indicate a transition to predominantly C_4_-based diets with no concurrent changes in trophic positions as inferred from bulk tissue nitrogen isotope (δ^15^N) values (*2*, *20*). These patterns are consistent with increased reliance on maize and the incorporation of maize-eating animals into human diets. Although preservation challenges limit the availability of archaeological fauna in the Neotropics of Mesoamerica earlier than 3000 B.P., maize provisioning likely began before this time. After 2400 B.P., people continued adapting subsistence strategies by increasing direct consumption of maize (*20*, *31*) and/or feeding it to animals (*29*). By 2000  B.P., zooarchaeological data reveal increased investment in species that often fed near agricultural fields where maize cultivation influenced their diets (*32*). Moreover, intentional or incidental feeding of wild animals may have increased their maize consumption, supplementing the maize they opportunistically ate from agricultural fields and possibly at levels higher than would have occurred without human involvement (*33*, *34*). These combined strategies of consuming maize and incorporating maize-eating animals into the diet highlight the innovative direct and indirect ways early communities optimized the nutritional value of maize to address its associated nutrient deficiencies as they transitioned from forager-based economies to those centered on farming.

**Fig. 1.**
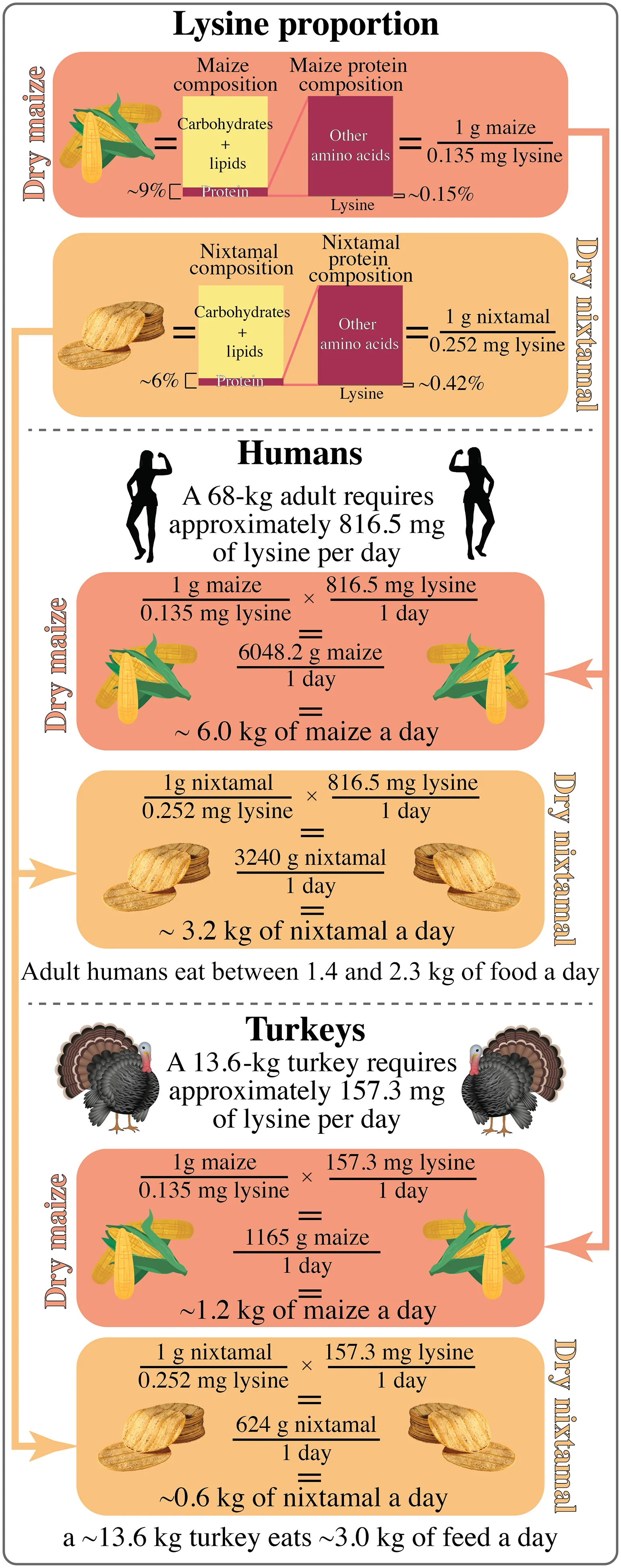
Human and turkey maize and nixtamalized maize daily requirements. (**Top**) Simplified macromolecular composition of dry maize and nixtamalized maize, highlighting their protein content and lysine deficiency. (**Middle**) Calculations illustrating the quantities of dry maize and nixtamalized maize required for an average 68-kg adult to eat to meet daily lysine requirements. (**Bottom**) Calculations showing the amount of dry maize and nixtamalized maize needed to meet the daily lysine requirements of a 13.6-kg turkey, emphasizing the role of C_4_-derived lysine trophic magnification in dietary strategies.

The nutritional limitations of a diet dominated by maize are well documented, particularly its deficiency in lysine (*5*), an essential amino acid crucial for human health. Lysine deficiency can cause severe health issues, including protein-energy malnutrition, stunted growth, impaired metabolism, defective connective tissue, and anemia (*35*), resulting in diminished quality of life, fecundity, and longevity. While maize is ~75% carbohydrates and ~9% protein by dry mass (*36*), lysine constitutes only 0.15% of its protein (*37*), requiring an average adult (~68 kg) to consume >6 kg of dry maize daily to meet lysine requirements (*38*), a physiologically unfeasible amount (Fig. 1 and table S4). Nixtamalization, an alkaline-based preparation method (*6*), reduces maize’s protein concentration but nearly triples bioavailable lysine (0.42%) (*39*), greatly improving its protein efficiency ratio (*40*). Although the origins of nixtamalization are unknown, this process likely contributed to the utility of maize as a staple food. Yet, nixtamalized maize consumption alone cannot be used to meet human lysine needs, necessitating supplementation from lysine-rich sources most reliably obtained through animal protein or lysine-rich plants (Fig. 1). Unlike humans, most herbivores and small-bodied omnivores can meet their lysine needs by consuming maize or nixtamalized maize. For example, an average 20-week-old turkey weighing 13.6 kg would require just 1.2 kg of maize (40% of its daily diet) or 0.64 kg of nixtamalized maize (21% of its daily diet) to meet lysine requirements (*41*). This raises the possibility that humans may have met their lysine needs by consuming animals provisioned with maize, a pathway we explore using amino acid δ^13^C analysis (*42*) integrated with nutritional modeling. This innovative technique allows us to trace and quantify individual essential amino acids, such as lysine, to their primary producer origins (e.g., maize) and explore how emergent and established agricultural communities mitigated potential nutritional deficiencies through the integration of plant cultivation and animal provisioning.

The δ^13^C values of bulk collagen represent a composite signal of its constituent amino acids, reflecting a mixture of routed dietary protein with those synthesized during metabolism (*21*). This mixture of compounds derived from exogenous sources (diet) and endogenous processes (metabolism) can obscure the relative contributions of plant and animal protein to tissue synthesis, limiting the resolution of bulk collagen δ^13^C analysis for identifying macromolecular origins in consumer tissues (*42*). Amino acid δ^13^C analysis overcomes this limitation by isolating individual compounds and linking their isotopic composition to specific biosynthetic pathways (*43*). Essential amino acids that animals cannot synthesize are directly routed into tissues with negligible isotopic fractionation, providing an isotopic fingerprint of their primary producer origins (*42*). Nonessential amino acids are also more efficiently routed directly from the diet but can be synthesized by animals using carbon from other dietary macromolecules (carbohydrates or lipids), resulting in isotopic fractionation that can obscure the original carbon origins (*44*, *45*). The extent of routing versus de novo amino acid synthesis depends on dietary protein quantity and quality, as well as the physiological state of the consumer (*46*, *47*). Animal-derived proteins are particularly well suited for isotopic tracing via amino acid δ^13^C analysis because their essential amino acid concentrations and relative abundances closely mirror those in consumer tissues (*48*, *49*), resulting in greater assimilation efficiency than dietary plant proteins (*50*). This efficiency increases their contribution to proteinaceous tissues such as bone collagen (*51*). Together, these dynamics give rise to what we term trophic magnification, defined here as the increasing proportion of an isotopically distinct nutrient (e.g., C_4_-derived lysine) in consumer tissues through trophic transfer, even when the total concentration of that nutrient does not increase across trophic levels. By analyzing the δ^13^C values of essential amino acids from archaeological remains and integrating these data with amino acid concentrations of key foods, this compound-specific approach provides a powerful lens to reconstruct macronutrient contributions to diet (*52*), allowing for detailed insights into how ancient populations adapted to changing ecological and demographic conditions that affected resource availability while maintaining nutritional balance.

In this study, we treat animal provisioning as one component of broader animal management strategies which encompass a continuum of human-animal interactions from opportunistic exploitation to more sustained, structured relationships. We use amino acid δ^13^C analysis to examine human nutrition during the transition to agriculture in southern Belize, where human activity has shaped the landscape into a mosaic of managed tropical forest and agroforestry plots since the Early Holocene (*53*, *54*). We present evidence from amino acid δ^13^C analysis of directly dated human remains spanning 6100 to 1100 B.P. excavated from the rock shelter sites Mayahak Cab Pek (MHCP) and Saki Tzul (ST) and the Classic Maya urban centers Uxbenká (UXB) and Ix Kuku’il (IK), alongside modern plants and turkeys. Using concentration-dependent Bayesian mixing models based on δ^13^C values of lysine, we (i) trace the incorporation of maize-eating animals into Neotropical diets during the agricultural transition, (ii) assess when maize-eating animals likely became a regular dietary component for humans, and (iii) estimate the quantities of maize, maize products, and maize-eating animal protein in human diets reliant on maize. Our findings shed light on the integration of maize cultivation and human-animal provisioning relationships in shaping subsistence economies and human nutrition during the Middle and Late Holocene in the Neotropics. By exploring how ancient populations mitigated the risks of nutritional deficiencies in the face of a changing world, our study deepens our understanding of the complex interplay between food security, sustainability, and human health, offering perspectives that resonate beyond the past.

## RESULTS

### δ^13^C values

We generated δ^13^C values of bulk bone collagen and five constituent essential amino acids (lysine, leucine, isoleucine, phenylalanine, and valine) for all ancient humans (*n* = 39), modern plants (*n* = 48), and modern turkeys (*n* = 5) (data S1 and table S2). δ^13^C values for modern samples were adjusted for the Suess effect to ensure comparability with prehistoric values due to a change in the carbon isotopic composition of the atmosphere caused by the burning of fossil fuels (*55*). The plant dataset included native C_3_ wild and domestic species as well as local maize varieties (C_4_; table S2). All bulk collagen samples yielded atomic C:N ratios within the accepted range of 2.8 to 3.5, confirming preservation and suitability for isotopic analysis (data S1) (*56*, *57*).

Bulk δ^13^C values of modern C_3_ plants ranged from −36.5 to −25.4‰ (mean = −30.5 ± 2.7‰), while maize (C_4_) values ranged from −13.8 to −11.5‰ (mean = −12.1 ± 0.8‰). These bulk values are presented to demonstrate the range of variation in our modern plant samples. The essential amino acid means ± SD δ^13^C values used in subsequent analyses are reported in table S2.

**Fig. 2.**
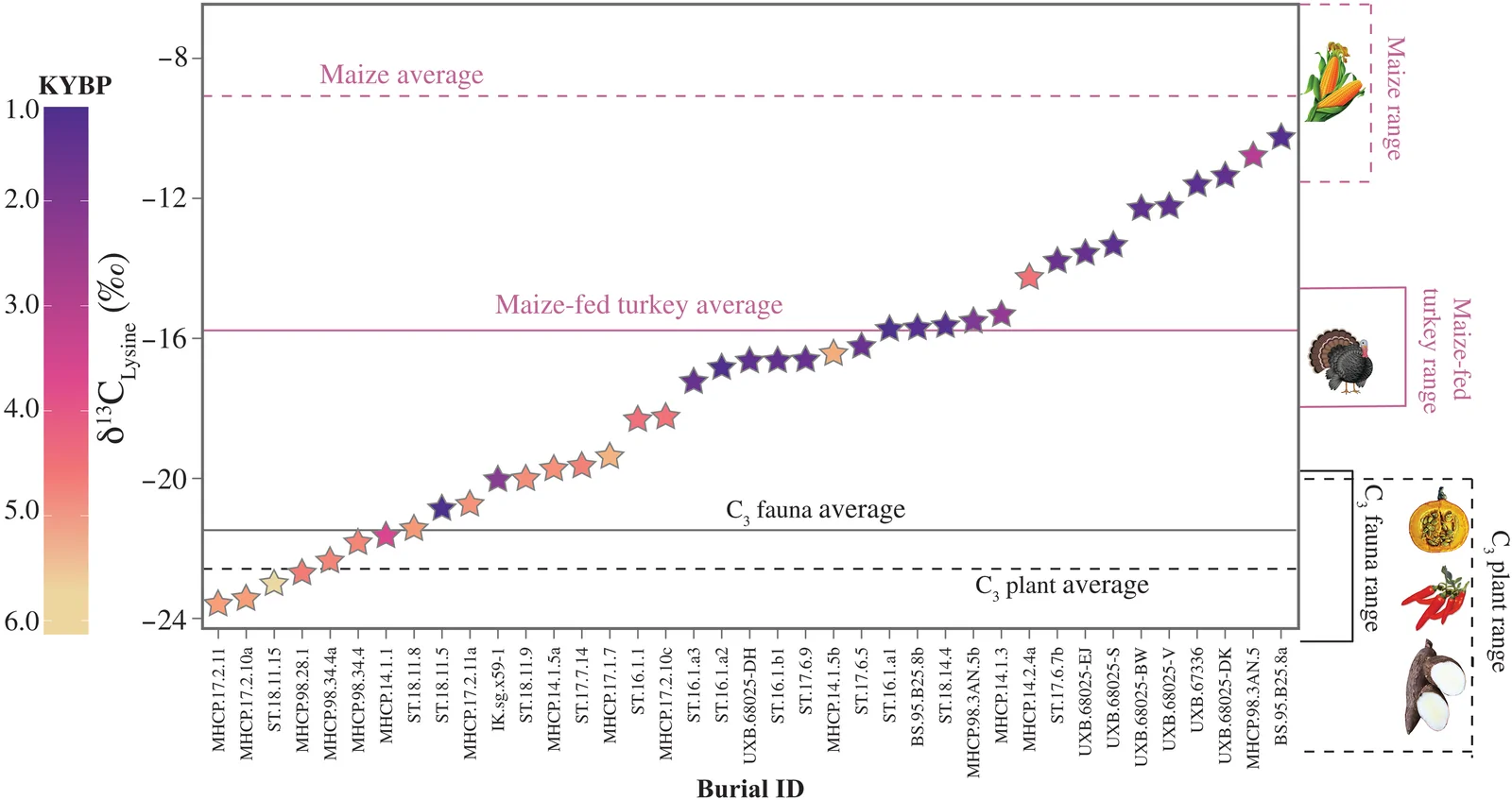
Lysine δ^13^C values of individuals, ordered from lowest to highest, compared with the isotopic ranges of C_3_ plants, C_3_-consuming fauna, C_4_ plants, and C_4_-consuming fauna. Each point represents an individual human, while solid and dashed lines denote comparative faunal and plant comparative references means and ranges only. The individuals are color-coded on the basis of their calibrated radiocarbon ages (in thousands of years B.P.) to illustrate chronological trends. Horizontal lines mark the average lysine δ^13^C values for modern maize-fed turkeys, modern maize, and C_3_ plants/fauna, contextualizing dietary contributions. This figure highlights the variation in lysine δ^13^C values, emphasizing the incorporation of maize-consuming animal protein and maize-derived products in diets over time, particularly during periods of agricultural intensification. Individuals with lysine δ^13^C values above the maize-fed turkey mean suggest elevated C_4_-derived lysine contributions, potentially due to the consumption of animals with diets richer in maize than our modern proxies. This variation reflects the dynamic interplay between maize cultivation, animal management, and dietary strategies across the study period.

Bulk collagen and essential amino acid δ^13^C values of humans are consistent with a distinct dietary transition over the study period (6100 to 1100 B.P.) from C_3_-based to C_4_-based protein sources (bulk δ^13^C ≈ −22.0 to −8.2‰; Fig. 2, fig. S1, and data S1). From 6100 to 3000 B.P., individuals (*n* = 18) shifted from predominantly C_3_-derived protein to C_4_ sources, with considerable variation between individuals (bulk δ^13^C ≈ −21.0 to −13.1‰) observed from 4700 to 3000 B.P. (*n* = 9). From 3000 to 1100 B.P., individuals (*n* = 21) obtained most of their protein through a C_4_ pathway with some variation among individuals (bulk δ^13^C ≈ −13.5 to −8.2‰), suggesting differential access to dietary resources.

δ^13^C values of modern turkeys are consistent with a mixed C_3_/C_4_ diet typical of maize-fed animals (*58*) with bulk collagen δ^13^C values ranging from −12.7 to −10.5‰ (mean = −11.9 ± 0.9‰) and lysine δ^13^C values ranging from −16.0 to −12.7‰ (mean = −13.9 ± 1.3‰; data S1).

### Stable isotope mixing models

A pooled mixing model using δ^13^C values of five essential amino acids (lysine, leucine, isoleucine, phenylalanine, and valine) suggests that, in addition to evidence for C_4_ protein consumption by 6100 B.P., there was a marked increase in mean (±SD) C_4_ protein consumption after 3000 B.P. (stable isotope mixing models were run in MixSIAR; full specifications including priors, Markov chain Monte Carlo (MCMC) parameters, and error structures are provided in Supplementary Text). Individuals dated from 6100 to 3000 B.P. (*n* = 18) derived 22.8 ± 12% of their protein from C_4_ sources, which increased to 79.6 ± 6.1% after 3000 B.P. (*n* = 21). Variation in C_4_-derived protein consumption was more pronounced among earlier individuals, ranging from 10.7 ± 4.9% to 53.7 ± 8.7%. By 3000 B.P. and later, C_4_-derived protein consumption ranged from 60.9 to 87.3% (fig. S2).

**Fig. 3.**
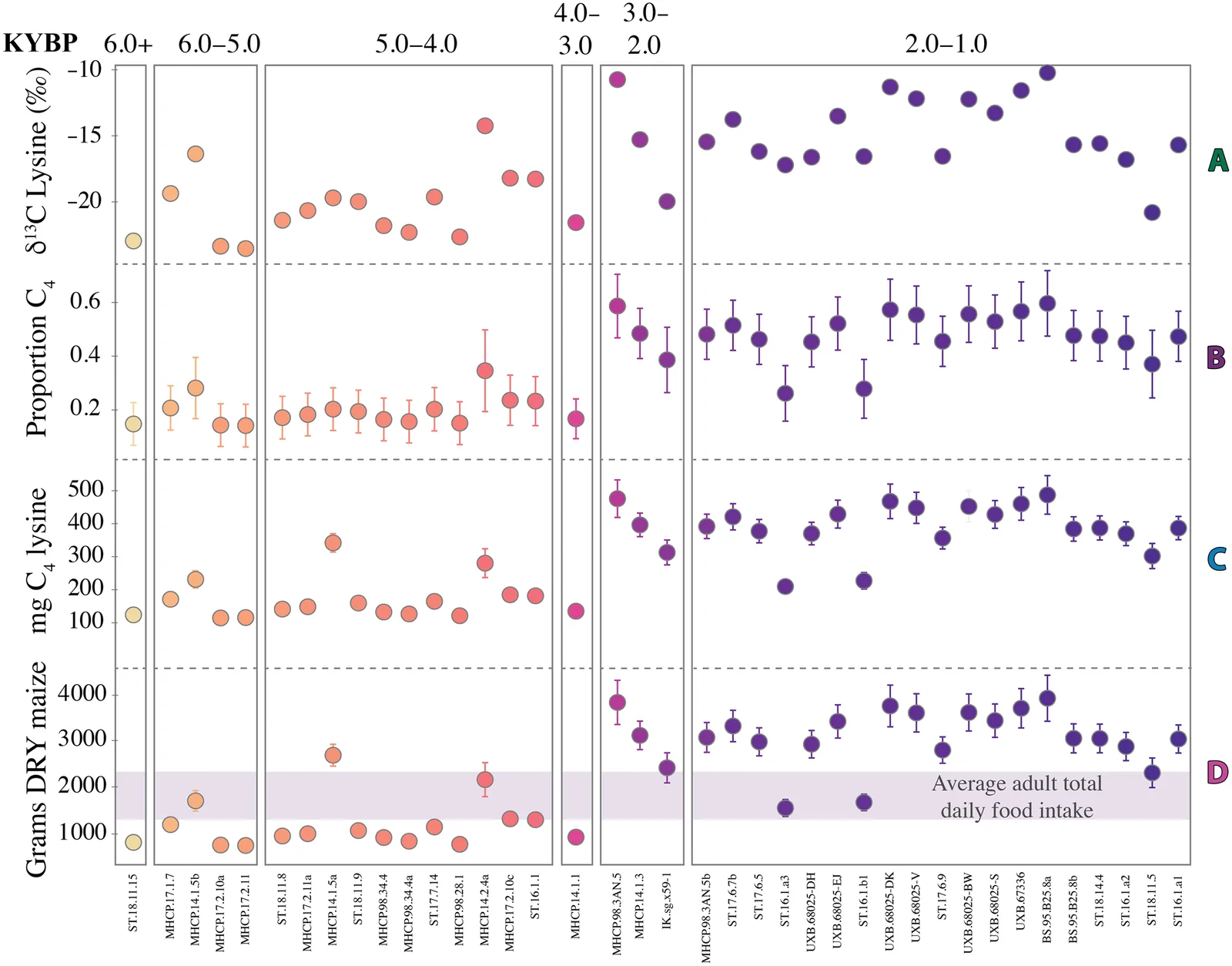
Lysine-based dietary calculations and results across time. Individuals are organized chronologically by calibrated radiocarbon age (B.P.) and grouped into 1000-year time bins. (**A**) Individual δ^13^C_lys_ values reflecting lysine isotopic compositions. (**B**) Proportion of lysine derived from C_4_ sources, calculated using MixSIAR mixing models with 95% confidence intervals. (**C**) Estimated daily intake of C_4_-derived lysine (milligrams) per individual, adjusted for body weight and age categories. (**D**) Daily grams of dry maize required to meet each individual’s estimated C_4_-derived lysine needs, contextualized within the average adult’s total daily food intake (highlighted in lavender).

**Fig. 4.**
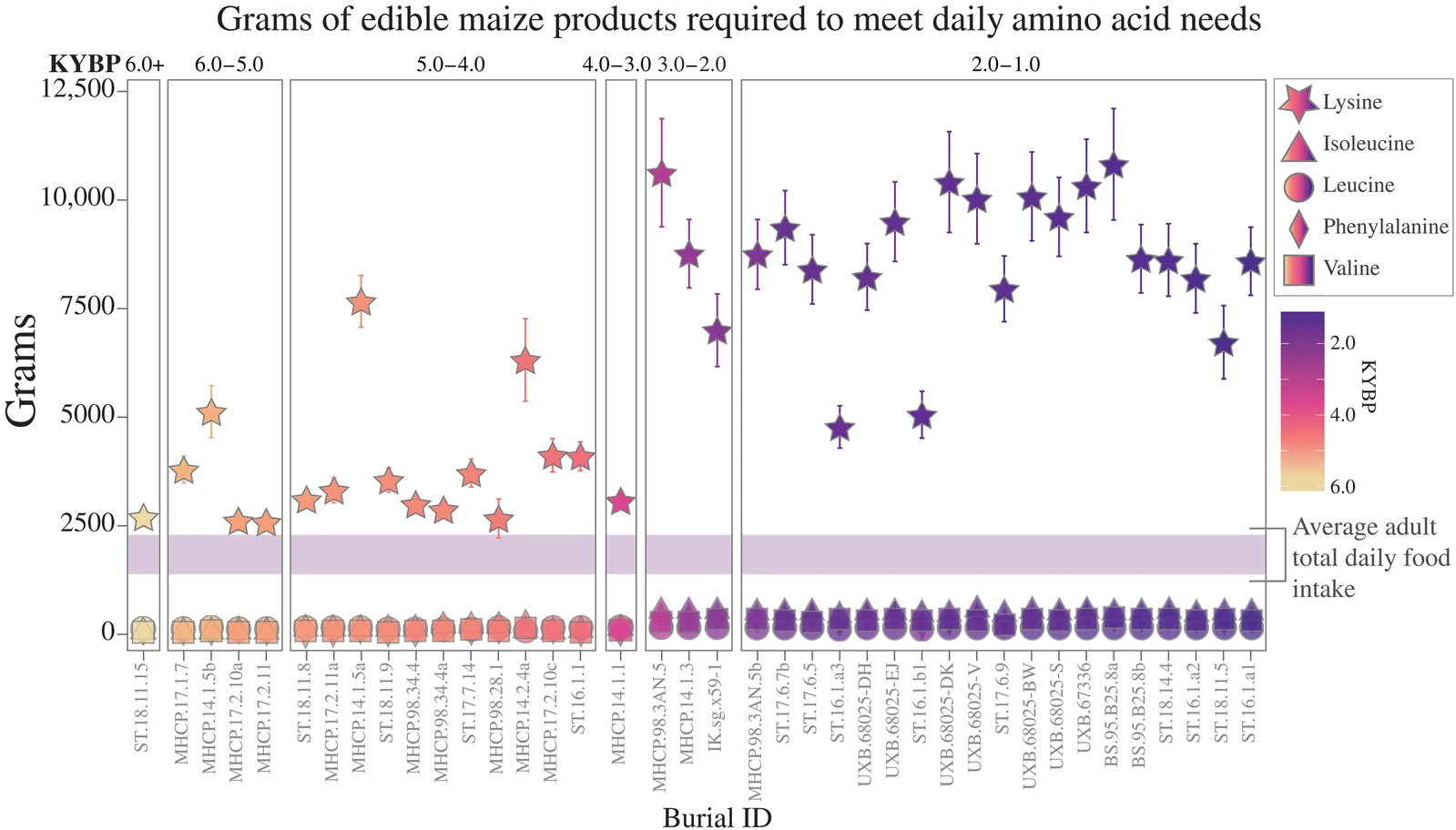
Comparison of the daily amount of raw dry maize each individual would need to consume to meet essential amino acid requirements. The chart displays the estimated maize intake required for lysine, isoleucine, leucine, phenylalanine, and valine, with individuals ordered chronologically and grouped into 1000-year time bins. While theoretical requirements for all essential amino acids except lysine could be met by maize consumption, the lysine deficiency in maize necessitated alternative dietary supplementation, emphasizing the importance of maize-consuming animal protein in early diets. The shaded lavender region indicates the average total daily food intake for adults, underscoring the improbability of relying solely on maize for lysine needs.

To further evaluate how contributions varied by amino acid, we also ran separate mixing models for each of the five essential amino acids analyzed, thus incorporating concentration dependence to account for their differing abundances in maize protein. These amino acid–specific models similarly show a shift from C_3_ to C_4_ carbon sources over time (Fig. 3 and fig. S3) but demonstrate distinct patterns in the C_4_-derived proportions of individual amino acids in human collagen, likely reflecting their concentrations in maize protein. For example, leucine, the most abundant essential amino acid in maize (18.7% of total protein), showed C_4_ contributions to human collagen ranging from 46.1 to 98.2%. In contrast, lysine, the least abundant in maize protein (0.15% of total protein), ranged from 14.1 to 59.6% (data S1). The differences in modeled C_4_-derived proportions across the study population underscore how the relative abundance of each amino acid in maize can shape its isotopic signal in consumer collagen and further highlight lysine as a limiting nutrient (Fig. 4).

### Dietary implications: C_4_ amino acid requirements

Using the C_4_ proportions obtained from mixing models, we calculated the daily amino acid requirements derived from C_4_ sources based on estimates of age and weight for each individual (data S1 and table S3) to determine the quantity of dry maize necessary to meet the C_4_-derived amino acid requirements. In addition to our basal models, we also performed a series of sensitivity analyses to confirm our results and subsequent interpretations. All results of our sensitivity analyses support the conclusions from our base models (Supplementary Text and data S1). The daily intake of dry maize to meet lysine requirements ranged from 853 ± 67 g to 3656 ± 431 g, an unrealistic amount. In contrast, the daily intake of dry maize to meet leucine requirements ranged from 26 ± 4 to 60 ± 9 g (Fig. 4). Our results underscore lysine’s limiting role among all the essential amino acids analyzed and highlight the need for alternative C_4_-based sources of lysine to account for lysine δ^13^C values, even in the earliest individuals (6100 B.P.).

### Alternative lysine sources: Nixtamalized maize and maize-eating animal meat

**Fig. 5.**
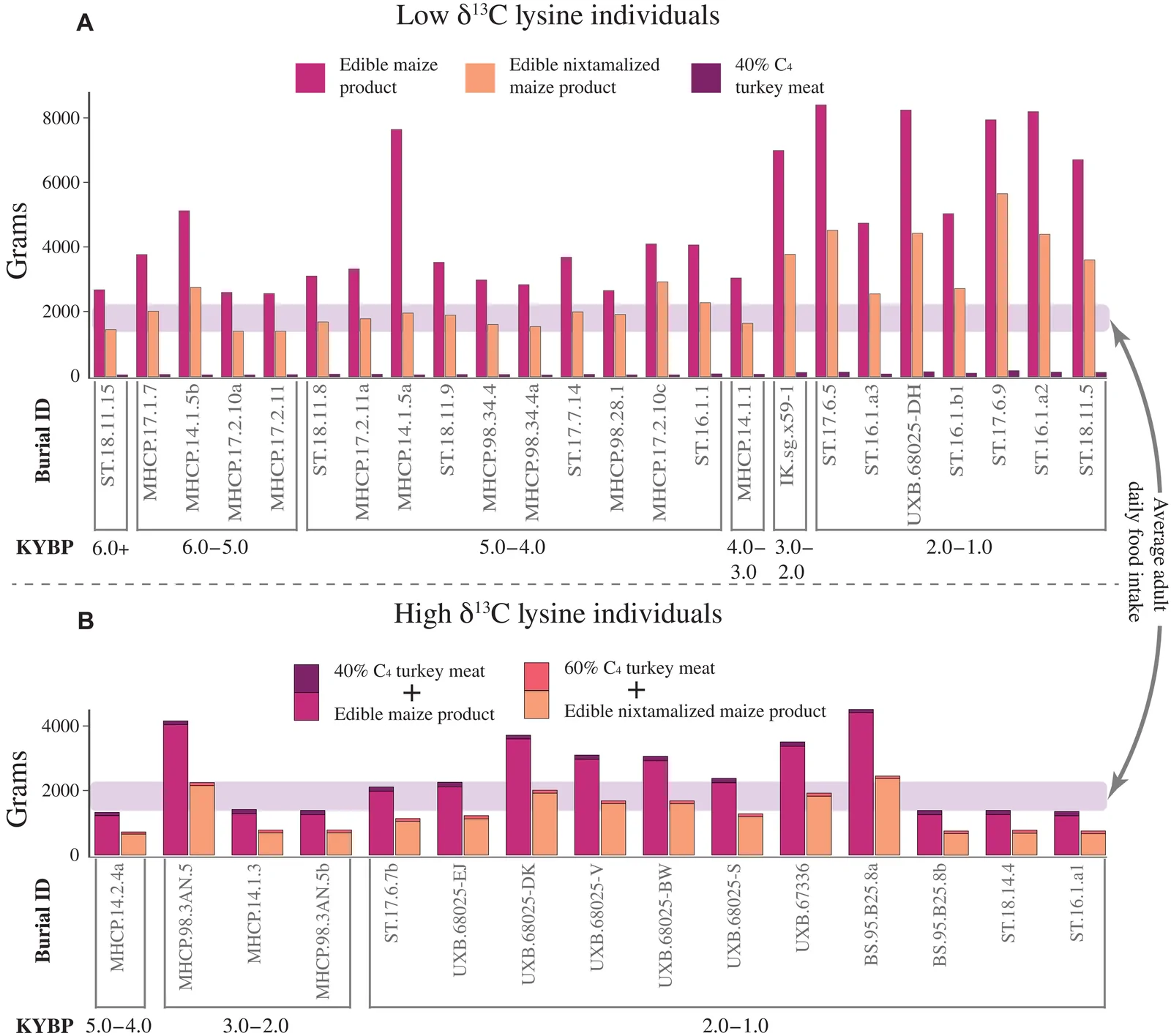
Modeled dietary scenarios for individuals with low and high δ^13^C lysine values. Panels show model outputs for individuals with relatively low (**A**) and high (**B**) δ^13^C lysine values. The modeling scenarios differ because these groups required distinct dietary combinations to reproduce their observed lysine δ^13^C values. Individuals in (B) could not be reconciled with lower C_4_ animal baselines alone, necessitating combinations of C_4_-derived lysine from maize-eating animals and edible maize products, whereas such scenarios were neither required nor informative for individuals with lower lysine δ^13^C values. Differences between panels, therefore, reflect biologically and nutritionally distinct possible solutions. Individuals are arranged chronologically to illustrate diachronic dietary trends. Lavender shading indicates the average total daily food intake range of adults (1400 to 2300 g), demonstrating the impracticality of meeting lysine needs through maize or nixtamalized maize alone and emphasizing the importance of animal-derived protein. (A) Individuals with lower δ^13^C lysine values than the modern turkey mean. Bars represent estimated daily intake of maize, nixtamalized maize, and 40% C_4_-fed turkey meat required to meet modeled C_4_-derived lysine needs, assuming contributions from a single source. (B) Individuals with higher δ^13^C lysine values than the modern turkey mean. Two modeled potential food combinations are shown: (i) maize combined with 40% C_4_-fed turkey meat and (ii) nixtamalized maize combined with 60% C_4_-fed turkey meat. These combinations represent nutritionally constrained scenarios that can be used to reconcile elevated lysine requirements.

To evaluate other potential sources of C_4_-derived lysine, we calculated the amounts of dry nixtamalized maize and maize-fed turkey meat required to meet daily C_4_-derived lysine needs for each individual. The dry weight of nixtamalized maize necessary to provide sufficient lysine ranged from 457 ± 253 g to 1931 ± 389 g. While less nixtamalized maize is required for each individual, converting it into edible products such as tortillas or tamales would require consumption of 1142 ± 232 g to 4827 ± 972 g per day, an amount also considered unrealistic (Fig. 5A).

To estimate the potential contribution of maize-eating animals, we used modern turkeys as a proxy by first establishing parameter bounds by running a two-source (C_3_ and C_4_ plants) mixing model for modern turkeys, which yielded two scenario values (~60 and ~40%) for C_4_-derived lysine. Human individuals were then categorized into two groups based on whether their lysine δ^13^C values were above (*n* = 15) or below (*n* = 24) the mean value for turkeys (−15.9‰; data S1).

Individuals with lysine δ^13^C values lower than the turkey mean (*n* = 24; 6100 to 1100 B.P.) would have required 13.3 ± 7.4 g to 54.8 ± 11.2 g of turkey meat daily (the equivalent to a chicken wing), assuming a 40% C_4_-derived lysine content. Under the higher 60% C_4_ baseline, the required amount drops to 8.9 ± 4.9 g to 36.5 ± 7.5 g daily (Fig. 5A). These values are derived from modern free-ranging turkeys and are used as conservative parameter bounds for scenario-based calculations, not as fixed empirical measurements of ancient animal diets.

**Fig. 6.**
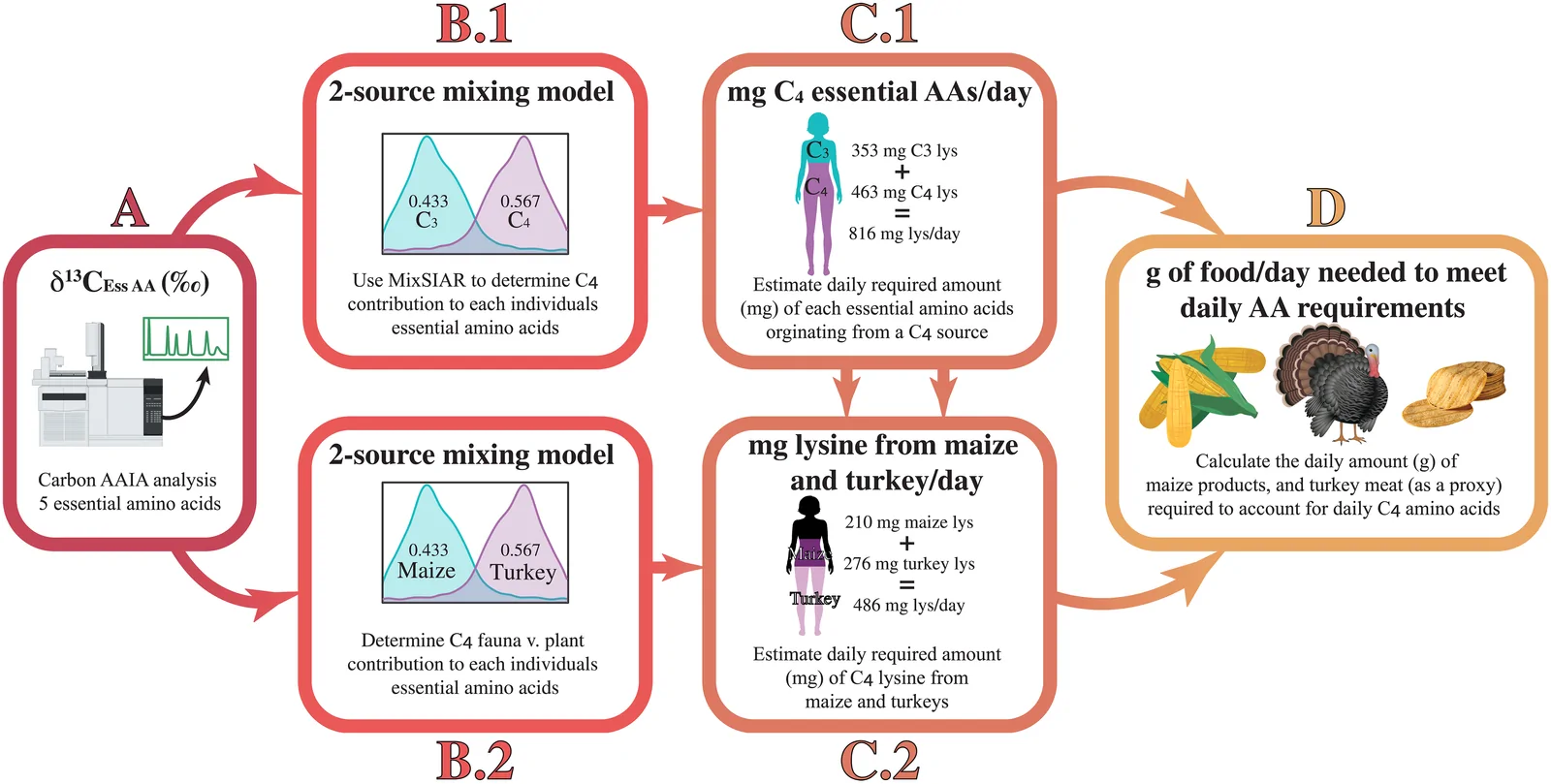
Analytical framework for calculating dietary contributions of maize-based foods and maize-consuming animals to individual lysine δ^13^C values. (**A**) Carbon amino acid isotope analysis (AAIA), focusing on lysine, leucine, isoleucine, phenylalanine, and valine. (**B.1**) Two-source stable isotope mixing models estimating the proportion of C_4_ versus C_3_ contributions to individual essential amino acids, based on dietary sources (e.g., maize and maize-consuming animals). (**B.2**) Additional mixing model steps applied to individuals with lysine δ^13^C values exceeding modern maize-fed turkeys, estimating contributions of C_4_-derived lysine from maize and maize-fed animals. (**C.1**) Calculations of daily milligram requirements of C_4_-derived essential amino acids for each individual, adjusted for average body weight and age category. (**C.2**) Detailed lysine-focused calculations estimating the proportion of lysine obtained from maize versus maize-fed animal protein. (**D**) Final dietary modeling outputs estimate the grams of maize, nixtamalized maize, or turkey meat (or a combination thereof) required to meet daily dietary lysine requirements. This integrative approach elucidates the dietary strategies ancient populations used to overcome maize’s lysine deficiency through trophic magnification in maize-consuming animals.

For individuals with lysine δ^13^C values higher than the turkey mean (*n* = 15; 14 dated between 1110 and 2700 B.P., one dated 4325 B.P.), we ran an additional two-source mixing model using mean (±SD) lysine δ^13^C values from C_4_ plants and turkeys. These models allowed us to estimate the milligrams of C_4_-lysine potentially derived from C_4_ animal protein versus C_4_ plant protein sources and calculate the corresponding quantities of turkey meat, dry maize, or dry nixtamalized maize required to meet lysine needs. The proportion of lysine derived from maize-eating animals ranged from 85.7 ± 12.9% to 59.2 ± 21.9%, while the proportion from C_4_ plants ranged from 40.8 ± 21.9% to 14.3 ± 12.9%. The total C_4_-derived lysine ranged from 329.5 ± 4.6 mg to 288.0 ± 12.8 mg from maize-eating animals and 55.0 ± 4.5 mg to 198.5 ± 12.8 mg from C_4_ plants. These lysine contributions translate to 38.1 ± 0.5 g of turkey meat (40% C_4_ content) or 25.4 ± 0.5 g (60% C_4_ content) combined with 407.2 ± 33.8 g to 1470.4 ± 94.7 g of dry maize or 218.2 ± 18.1 g to 787.7 ± 50.7 g of dry nixtamalized maize (Figs. 5B and 6).

## DISCUSSION

Our concentration-dependent paleodiet models suggest that by 6100 B.P., Neotropical communities were likely incorporating protein derived from maize-eating animals into their diets. This practice may have helped enable the sustained reliance on maize by ancient agricultural societies in Mesoamerica, highlighting the innovative strategies past populations used to address nutritional challenges associated with the transition to agriculture. Our isotopic data, when evaluated using concentration-dependent models and macronutrient constraints in common foods, are most consistent with the hypothesis that the individuals analyzed regularly consumed protein from maize-eating animals, suggesting that strategies involving regular access to maize-eating animals may have been in place in Mesoamerica at least 4000 years before the first direct evidence of domesticated animals (~2200 B.P.) in regional zooarchaeological assemblages (*10*).

Regional paleoecological and paleobotanical records indicate that, aside from trace amounts of amaranth (*Amaranthus* spp.) in Holocene-dated palynological cores (*59*, *60*), maize was the only abundant C_4_ plant in the study region during the periods considered (*2*). As a result, any substantial C_4_-derived carbon in human collagen is most parsimoniously attributed to maize, with minor contributions from other C_4_ plants remaining possible but unlikely as drivers of the observed patterns. Furthermore, since maize is a seasonally grown crop harvested and preserved for year-round consumption, and because bone collagen is a metabolically active tissue that integrates dietary inputs over many years, the observed patterns are difficult to reconcile with occasional or seasonal reliance on maize-eating animals. Instead, our results align more with an active, sustained strategy to offset the nutritional limitations of maize-dependent subsistence by maintaining regular access to animals that ate maize, possibly through direct provisioning as a reliable source of protein. Our findings raise the question of the extent to which maize was cultivated and ultimately domesticated for human consumption rather than for animal provisioning.

Our modeled proportions of C_4_-derived lysine represent only a fraction of each individual’s total daily lysine intake and overall diet. The remaining lysine was obtained primarily from C_3_ sources, including wild, cultivated, and domesticated foods, depending on the time period. For instance, even those with the lowest modeled proportions of C_4_-derived lysine (~15%) would have sourced the majority (~85%) of their lysine through C_3_ pathways, reflecting diverse combinations of C_3_ plants and animals consuming those plants within mixed subsistence systems. Our models do not imply that maize-eating animals replaced these broader dietary foundations. Instead, they show that even this modest C_4_-derived lysine fraction is unlikely to have been met solely through the direct consumption of maize or nixtamalized maize under realistic physiological assumptions, particularly given the substantial intake of other foods required to meet the remaining C_3_-derived lysine and other nutrient needs. Under these constraints, trophic magnification via the consumption of maize-eating animals is the most plausible explanation for the presence of C_4_-derived lysine, which aligns with paleoecological evidence identifying maize as the dominant C_4_ plant in the region. Therefore, animal provisioning should be understood not as the primary or exclusive source of dietary protein but as a mechanism that enabled small yet nutritionally important contributions of maize-derived lysine within diverse and adaptable food systems.

To evaluate the robustness of our interpretations, we performed sensitivity analyses by adjusting trophic discrimination factors (TDFs) by ±0.5‰, estimated body weights by ±20%, and daily lysine needs by ±20% (Supplementary Text and data S1). Across all parameter combinations, the models’ qualitative outcomes remained unchanged: The modeled C_4_-derived lysine proportions cannot be reconciled by maize or nixtamalized maize alone under our physiological constraints. Even with the most conservative assumptions that minimized C_4_-derived lysine needs (e.g., +0.5‰ TDF, −20% body weight, and −20% daily mg/kg of lysine), consuming enough edible maize or nixtamalized maize products to match the modeled C_4_-derived lysine proportions still would have required sustained intake levels approaching the upper bounds of realistic daily diets. This constraint is even more pronounced for individuals from earlier periods, associated with the low-yield maize varieties present on the landscape early in the maize-domestication process (*61*). Together, these sensitivity tests support and reinforce the inference that trophic magnification via maize-eating animals remains the most plausible and cautious explanation for the presence of C_4_-derived lysine in human collagen within this study system.

Although direct evidence is lacking, the possibility that animal provisioning influenced the trajectory and investment in maize cultivation carries substantial implications. It suggests that the nutritional needs of humans and the animals they depended on may have shaped both plant and animal domestication processes, creating a mutualistic system in which maize and maize-eating animals reinforced each other’s value. While this hypothesis requires further testing, it emphasizes the importance of viewing human-animal provisioning relationships not just as a consequence but as a potential driver of early agriculture. This dynamic would have operated through trophic magnification, in which C_4_-derived lysine became concentrated in humans via animals that consumed or were provisioned with maize. Over time, such practices may have become increasingly important components of subsistence strategies, consistent with coevolution between maize cultivation and human-animal provisioning systems, which likely served as an adaptive nutritional solution for ancient populations.

An early sign of this synergistic relationship appears by 4300 B.P., when some individuals’ modeled values indicate higher proportions of maize-eating animal protein in their diets (Fig. 5B), suggesting more intensive forms of animal provisioning strategies such as direct feeding with maize and/or nixtamalized maize, potentially at levels exceeding those observed in contemporary Maya communities. Overall, these patterns indicate that innovations in early farming systems could have enabled people to meet their nutritional needs despite social and ecological constraints. A growing dependence on maize, including direct consumption and (intentional or incidental) animal foddering, would have marked a pivotal transition in subsistence that laid the groundwork for Classic Maya agricultural systems. These long-term strategies for nutritional resilience remain relevant today in the context of food security and sustainability. The following sections explore the potential implications of integrating animal provisioning into ancient Neotropical diets, as suggested by our model outputs; quantify the dietary role of maize and maize-eating animals using modeled scenarios; and examine the limits and broader implications of these patterns for understanding resilience in early food systems.

### Integration and temporal trends of animal provisioning into Neotropical diets

#### Early reliance on maize-eating animals

Our results are most consistent with maize-eating animals being regularly incorporated into Neotropical diets by 6100 B.P. and suggest that their dietary role in relation to maize cultivation and provisioning strategies shifted over time. Even individuals with the lowest modeled proportions of C_4_-derived lysine show evidence of trophic magnification. For example, the individual with the lowest estimated proportion (14.1%) of C_4_-derived lysine (MHCP.17.2.11, 5070 B.P.) would have needed to consume an impractical ~3400 g of fresh maize kernels per day (~850 g dry weight equivalent) to achieve this proportion, under the physiological assumptions central to our models. This amount is nearly twice the average total daily food intake (1815 g wet weight) of an adult and excludes the additional food required to meet other nutritional needs.

Such dietary demands underscore why maize foddering, and thus supplementation with maize-eating animal protein would have provided a more feasible means of meeting lysine requirements in maize-dependent diets. While this finding aligns with global trends documenting the interaction between animal provisioning and plant domestication, it is unique to Mesoamerica, where the domestication of large animals was limited compared to other domestication centers due to the widespread Late Pleistocene extinction of ungulates (*62*). Instead, smaller animals such as turkeys, ducks, rodents, and deer were incorporated into subsistence systems to varying extents (*29*, *63*, *64*), ranging from purposeful feeding to indirect management such as garden or opportunistic hunting in and around maize fields (*33*, *34*), practices still observed in some contemporary Maya communities. Furthermore, the range of modeled C_4_-derived lysine proportions among early individuals (14.1 to 34.6%) suggests that the strategies for doing so were varied and context dependent.

Variability in the modeled proportions (14.1 to 34.6%) of C_4_-derived lysine among individuals (*n* = 14; 6100 to 4300 B.P.) in the earliest period of our study suggests that subsistence strategies were not uniform but rather influenced by factors such as individual or community specialization, differential access to cultivated and wild food sources, and/or variation in the timing of adopting different provisioning practices. On the basis of model results, some individuals appear to have relied more on C_3_-based resources, while others suggest greater dependence on maize-eating animal protein, possibly reflecting regional or social differences in maize cultivation and animal provisioning strategies. For example, the earliest individual (ST.18.11.15; 6093 B.P.) was estimated to have an ~15% contribution of C_4_-derived lysine, which could be the result of several scenarios such as (but not limited to): (i) consuming animal protein with the same proportion of C_4_-derived lysine, leading to direct routing of both C_4_ (15%)– and C_3_ (~85%)–derived lysine; in this case, the maize-eating animal would be the sole source of animal protein; (ii) a combination of direct maize consumption and trophic magnification through consuming maize-eating animals (*34*), suggesting a diet low in protein; or (iii) the individual’s diet included animal protein with a higher proportion of C_4_-derived lysine than their own but also included consumption of other C_3_ animal protein sources. On the basis of species diversity in regional diachronic archaeological faunal assemblages (*65*), the third scenario may be the most plausible, highlighting the integration of both wild resources and those more closely tied to organized structures in diets during this early period.

If the maize-eating animals were among the primary sources of animal protein for the earliest individuals in our record, then these animals likely had lower C_4_-derived lysine proportions than our contemporary turkey proxies, perhaps as low as ~10 to 15% maize. The minimum estimated amount of maize required to rear a small-bodied animal (e.g., turkeys) to maturity on a diet of 10 to 15% maize is ~2.1 to 3.2 kg of dry maize (~5400 to 8000 maize-derived Cal) per animal in addition to the other C_3_-resources. In return, such an animal would provide an estimated 8500 to 13,800 of consumable Cal, resulting in a net caloric excess of ~3100 to 5800 Cal when maize is used as a feed-to-food energy source.

Given the size of semidomesticated varieties of maize dating to 5000 to 6000 years ago, which had fewer kernels (estimated at around 24 per cob) and seed rows (*61*), it would have required ~875 to 1300 ancient cobs to produce enough feed for one animal with 10 to 15% C_4_-derived lysine. However, these estimates are based solely on kernels; in some contemporary Maya farming communities, the cob and kernels are combined, ground, and fed to animals. Ancient populations may have used similar techniques, using stalks, cobs, and kernels for fodder, further reducing the number of maize plants required to sustain a small-bodied animal. Furthermore, relying on early maize cobs for direct human consumption, even through nixtamalization, would have required greater energetic expenditure in cultivation, harvest, and processing than using the same cobs as fodder. This energetic imbalance further underscores why, even with improvements in lysine concentration and bioavailability, these individuals would likely still have needed complementary protein sources from maize-eating animals to obtain the observed lysine δ^13^C values, based on our modeled scenarios. These early potential feeding strategies illustrate a scalable, energetically viable system that would have allowed animals to be sustained on relatively modest amounts of maize.

#### Transition toward intensified animal provisioning strategies

Our model results suggest that by 4300 B.P., maize-based animal protein may have emerged as an increasingly important dietary component, a trend hinted at in previously published bulk collagen δ^13^C and δ^15^N values for these individuals (*2*, *20*). Together, these patterns are consistent with a transition toward more deliberate forms of animal provisioning. The presence of individuals with high modeled proportions of maize-derived lysine is more consistent with intentional animal provisioning than incidental exploitation of wild species that foraged opportunistically on maize. Through trophic magnification, such practices could have supplied lysine-rich protein, mitigating the nutritional limitations of maize and reinforcing its role as a dietary staple. Given the multiyear integration of dietary inputs represented in collagen, along with the elevated lysine δ^13^C values found in many individuals, this pattern is difficult to reconcile with occasional or seasonal consumption of maize-eating animals. At the same time, the presence of some individuals with lower modeled proportions of C_4_-derived lysine implies potential variability in the intensity of animal provisioning, with some communities likely maintaining more opportunistic or mixed strategies. The diversity in practices reflects a continuum between wild and managed relationships, capturing an important transitional stage in the development of Neotropical food systems.

#### Widespread integration of maize-eating animals

By 3000 B.P., Neotropical communities were highly reliant on maize (*20*), and our model results indicate a marked increase in maize-based animal protein consumption, averaging ~48% among individuals, suggesting a strong association between maize cultivation and animal provisioning strategies. The nutritional trade-offs of using maize as animal feed are particularly relevant during this later period. Although cultivating small cereal crops for animal feed at contemporary levels results in a net caloric deficit since the calories (Cal) required to rear an animal exceed those obtained from that animal, it yields a higher-quality food source with a more complete macronutrient composition, particularly regarding protein. For example, modern turkeys raised for consumption typically eat around 11.1 kg of maize in their lifetimes before harvest, constituting ~52% of their diet (*66*). The quantity of maize required to provision a modern turkey before harvest contains ~28,400 Cal from maize, including ~6000 g of carbohydrates, 350 g of fat, and 1000 g of protein, with only 1.5 g of lysine. Given that ~65% of a turkey is consumable (*67*), a 9-kg turkey yields 11,000 Cal, in the form of 4 g of carbohydrates, 440 g of fats, and 1,700 g of protein, including 250 g of lysine. Therefore, while raising turkeys on a 52% maize diet results in a net deficit of ~17,400 Cal, it increases the overall nutritional quality of the new food source, improving the essential amino acid profile and effectively balancing the potential for lysine deficiency in maize-dominant human diets.

### Estimating maize, maize products, and maize-eating animal protein consumption

Based on the lysine δ^13^C data, the diets of many individuals in our study are most consistent with the incorporation of both animal protein from maize-eating animals and unaltered or nixtamalized maize. Of the 39 individuals analyzed, 15 adults had lysine δ^13^C values higher than those of modern maize-fed turkeys. Most of these individuals date between 2700 and 1110 B.P., coinciding with increased reliance on maize as a staple food and fodder resource in the Maya region. However, evidence for maize-derived protein in one individual (4325 B.P.) predates the first direct evidence of domesticated animals in Maya archaeofaunal assemblages by ~2000 years but aligns with the period when maize varieties were productive enough to constitute a large portion of human diets (*68*). The return rate for maize during this later period (*61*) was likely higher than that of earlier varieties, further evidenced by maize’s increasing dietary importance. The date of this individual suggests that by 4300 B.P., communities may have used varied subsistence and possibly food-processing techniques, with some more directly engaged in maize-based agricultural practices and others likely relying more on wild resources and/or cultivated C_3_ plants.

For 9 of the 15 individuals with elevated lysine δ^13^C values, the amount of maize needed to meet daily C_4_-derived lysine estimates, even when combined with maize-eating animal protein, would still have exceeded the average adult’s daily total food intake. This makes it difficult to reconcile their lysine δ^13^C values with our modeled diet combinations alone, suggesting that, under these assumptions, the animals they consumed may have obtained C_4_-derived lysine contributions that exceeded those observed in our free-ranging proxy range (40% to 60% in our dataset). This interpretation is further contextualized through a comparison between our modern turkey bulk collagen δ^13^C data (mean = −11.9 to 0.9‰; data S1) and published domesticated turkey bulk collagen δ^13^C values (mean = −9.3 to 1.5‰) from four prominent Maya urban centers dating from the Late Preclassic to Postclassic periods (*29*), which indicate variability, and in some cases, higher maize inputs in archaeological populations. Together, these patterns are consistent with variability in animal provisioning intensity, potentially ranging from free-ranging to more structured feeding practices. Inferences that require values beyond our modern proxy bounds should be understood as scenario-based requirements necessary to reconcile the observed human lysine δ^13^C values within our model structure rather than as direct empirical measurements of ancient animal diets.

### Human-animal-plant interactions in the Neotropics since 6100 B.P.

The increased reliance on maize-eating animals suggested by our model results aligns with a sustained nutritional strategy capable of mitigating the challenges of a maize-dominant diet. Supplementing maize-based diets with maize-eating animal protein could have helped populations balance their macronutrient and amino acid needs while maintaining diverse subsistence practices and supporting long-term dietary stability. Our findings are consistent with a coevolutionary dynamic linking maize cultivation, its ongoing domestication, and increasingly structured human-animal provisioning relationships. Instead of replacing broader C_3_-based food systems, maize-eating animals may have provided a mechanism by which small but nutritionally critical amounts of maize-derived lysine could enter human diets. Such innovations may have facilitated intensified agricultural practices and reinforced the enduring reliance on maize in Neotropical subsistence economies. Within this framework, maize-eating animals had the potential to provide a dependable source of high-quality protein, enhancing nutritional resilience by buffering the risks associated with maize’s inherent lysine limitation.

### Limitations and other considerations

Our findings offer important insights into the interplay between maize agriculture and human-animal provisioning relationships; however, a few limitations warrant consideration. First, analysis of modern turkeys cannot provide an exact proxy for ancient feeding practices because past maize landraces and feeding strategies likely differed. Second, our estimates of daily dietary requirements are based on modern averages (table S3) (*38*), which may not fully reflect the metabolic demands of the studied individuals. Third, our models do not consider potential de novo synthesis of lysine by the human gut microbiome; however, recent work suggests that the gut microbial contribution to lysine in mammalian tissues is minimal relative to other essential amino acids (*69*). The use of other amino acids in concentration-dependent paleodiet models may be more strongly influenced by microbial synthesis and should therefore be approached with additional caution. Although contemporaneous faunal isotopic data would provide valuable contextual information, the modeling approach used here does not require a one-to-one correspondence between associated animal remains and human individuals as it is grounded in nutritional physiology rather than species-specific management histories.

The modeling framework presented here is tailored explicitly to ecological settings characterized by low biomass of endemic C_4_ plants. In regions where multiple C_4_ plants are abundant or where diverse foddering systems were in place, these specific models will not be applicable. Nevertheless, we view the framework developed here as scaffolding that can be adapted to other contexts by incorporating regionally appropriate palaeoecological constraints, nutritional requirements, and macronutrient routing assumptions. While our findings emphasize maize as the primary source of C_4_-derived protein, it is essential to consider alternative explanations. Some studies indicate that varieties of the C_4_ plant amaranth may have been cultivated alongside or instead of maize (*70*, *71*), which could explain elevated δ^13^C values. Amaranth protein is rich in lysine but low in leucine compared to maize and human essential amino acid requirements (*72*). Our mixing models show that, for all individuals, the proportion of leucine derived from a C_4_ source is substantially higher than that of lysine. If amaranth rather than maize had been the primary C_4_ protein source, we would expect the opposite pattern under our model assumptions. Furthermore, if both amaranth and maize contributed to C_4_ protein intake, the proportions of C_4_-derived leucine and lysine would be more balanced than our data indicate. The leucine-lysine contrasts, therefore, serve as complementary evidence to regional paleoecological records, reinforcing maize as the most plausible dominant C_4_ plant capable of producing the observed isotopic patterns. Directly measuring amino acid profiles in archaeological plant remains would offer valuable insights into past variability in C_4_ plant protein sources. However, such analyses are not feasible for the tropical lowlands due to poor organic preservation and would require a separate investigation beyond this study’s scope. Despite these limitations, the overall trends we observed provide important insights into the strategies used by early agricultural societies to achieve nutritional balance.

## MATERIALS AND METHODS

### Community consultations and ethics statement

The Institute of Archaeology, National Institute of Culture and History in Belize, issued permits for fieldwork, export, and analysis of human remains. The Forest Department of Belize also issued additional permits to conduct fieldwork in the Bladen Nature Reserve. All research permits were supported by our local partner, the Ya’axché Conservation Trust, a nongovernmental organization dedicated to preserving local biodiversity and cultural heritage. Fieldwork was conducted in collaboration with Ya’axché and members of local communities. Research findings are presented biannually at the Belize Archaeology Symposium, a public conference attended by members of many Belizean communities. In addition, research outcomes are shared yearly with descendant communities through events organized by Ya’axché. These annual community consultations feature presentations from various project members regarding the status of different research aspects, the distribution of educational materials in local languages, and feedback sessions designed to ensure that descendant communities have an active voice in shaping future research directions.

### Sample collection

Our study population (*n* = 39) includes 8 males and 8 females; the sex of 23 individuals could not be determined because of the fragmentary condition of their remains. Our sample is predominantly composed of adults (*n* = 34), with three infants and two juveniles. Age estimations of all human remains were performed osteologically using transition analysis on more complete individuals. For those represented by single elements, broad age categories of “adult” or “nonadult” were assigned. Sex was determined either osteologically or through genomic analyses (*73*).

Calculations of the quantities of different maize-based products were conducted using age-dependent models that incorporated assumptions about average body weights and nutritional requirements (see Supplementary Text for detailed methods and results, including sensitivity analyses in which we varied body weights, nutritional requirements, and TDFs). In addition, we analyzed five modern turkeys (*Meleagris gallopavo*) collected from areas near UXB and IK that were raised free-range and consumed a diet of wild forage subsidized with locally grown maize and nixtamalized maize products. They serve as proxies for herbivorous/omnivorous animals that may have contributed to human protein intake in the past, including through animal provisioning. We also collected 48 modern wild and domesticated plants near UXB, IK, MHCP, and ST for amino acid δ^13^C analysis.

All human remains are curated at the University of New Mexico Maxwell Museum of Anthropology under permits issued to KMP by the Government of Belize following formal consultations with local descendant communities in the region (*2*, *73*) and adhering to strict ethical guidelines and formal collaborations with communities and local organizations. Plants and maize-fed turkeys were collected between 2020 and 2024 from Santa Cruz, Belize (Toledo District), and the Bladen Nature Reserve. All plant samples included only local native wild and domesticated species. Collections and exports were undertaken under permits issued by the Belize Forest Department and the US Department of Agriculture.

### Stable isotope analyses

Long bone fragments were preferentially selected for collagen extraction. In cases of preservation constraints, we used other elements with comparable tissue turnover rates. We extracted and purified bone collagen using the Longin method (*74*) combined with ultrafiltration. Subsamples of bone weighing ~500 mg were demineralized via submersion in 0.5 M HCl for 36 hours at 5°C and then rinsed to neutrality in 18.2 MΩ·cm H_2_O. Following demineralization, all archaeological samples were treated with 0.1 M NaOH to remove residual humic acids and then rinsed to neutrality in 18.2 MΩ·cm H_2_O. Samples of modern turkeys underwent lipid extraction via submersion in a 2:1 chloroform:methanol solution for 72 hours and then rinsing with 18.2 MΩ·cm H_2_O. All bone collagen samples were gelatinized in a 0.01 M HCl solution for 24 hours at 70°C before lyophilization. Extracted human bone collagen samples were purified using Sartorius Vivaspin20, 30-kDa MWCO Polyethersulfone ultrafilters. All plant samples were dried at 45°C and homogenized with a mortar and pestle.

We report all isotope data in δ-notation using the equation δ^13^C or δ^15^N = 1000 * [(*R*_sample_/*R*_standard_) – 1], where the *R*_sample_ and *R*_standard_ represent the ratios ^13^C:^12^C or ^15^N:^14^N in the sample and standard. δ values are expressed relative to internationally accepted standards, Atmospheric N_2_ for δ^15^N and Vienna-Pee Dee Belemnite for δ^13^C. For bulk tissue δ^13^C and δ^15^N analysis, ~0.7 to 1.0 mg of extracted collagen and ~5 mg of plant material were packed into 3 mm–by–5 mm tin capsules. Bulk δ^13^C and δ^15^N values were measured using a Costech 4010 Elemental Analyzer (Valencia, CA) connected to a Thermo Scientific Delta V Plus isotope ratio mass spectrometer (Bremen, Germany) at the University of New Mexico Center for Stable Isotopes (UNM-CSI). Analytical precision was determined by calculating the mean within-run SD of a suite of plant and protein-based internal reference materials. All SDs were <0.2‰ for both δ^13^C and δ^15^N values. We also measured the weight percent C:N ratios of all ancient bone collagen samples for quality control. Atomic C:N ratios were calculated from weight percent elemental data, and samples with atomic ratios outside the range of 2.8 to 3.5 (*56*, *57*) were excluded from further analysis. All samples yielded >1% collagen by total bone mass. In addition, we calculated the weight percent C:N ratios for all plants as a proxy for protein content.

For amino acid δ^13^C analysis, 3 to 10 mg of extracted and purified bone collagen and 20 to 30 mg of homogenized plant material were derivatized following established protocols (*21*, *42*, *75*). All samples were hydrolyzed in 1 ml of 6 M HCl at 110°C for 20 hours. During hydrolysis, glutamine and glutamate are converted to glutamic acid, and asparagine and aspartate to aspartic acid (*42*, *76*). The hydrolysates were then dried under N_2_ gas in a heat block at 110°C. The resulting free amino acids were derivatized to *N*-trifluoroacetic acid isopropyl esters via two steps (*76*). The first derivatization involves esterification of the carboxyl group(s) using 1 ml of a 4:1 isopropanol:acetyl chloride solution, reacted at 110°C for 1 hour. The second derivatization is an acetylation of the amine group(s) using 1 ml of 1:1 trifluoracetic anhydride:dichloromethane solution, reacted at 110°C for 10 min. Each set of samples was derivatized alongside internal reference materials consisting of pure samples of all measured amino acids purchased from a supplier (Sigma-Aldrich).

δ^13^C values of each derivatized sample were measured on a Thermo Scientific Trace 1310 gas chromatograph system equipped with a 60 m–by–0.32 mm ID BPX5 x 1.0-μm column (SGE) and Isolink II combustion interface coupled to a Delta V Plus (*75*, *77*, *78*) isotope ratio mass spectrometer at UNM-CSI. All samples were measured in duplicate or triplicate, with every two samples bracketed by the internal reference material. SDs for all unknowns and reference materials were calculated across injections for each amino acid (table S1), and any sample with an SD >1‰ was excluded from further analysis.

### Radiocarbon analyses

Radiocarbon (^14^C) data were generated from bone collagen for all samples (see data S1 for a list of burials dated during our study) for which ages have not been published. Sample preparation was conducted at UNM-CSI, and ^14^C was measured via accelerator mass spectrometry (AMS) at the Pennsylvania State University Radiocarbon Laboratory. Bone collagen extraction and purification procedures followed the same protocols outlined above. We used the same criteria to assess collagen integrity, based on collagen % yields and C:N ratios, before AMS analyses.

All extracted bone collagen samples were converted to graphite for analysis via AMS. Approximately 2.4 mg of each sample was weighed into quartz tubes along with ~10 mg of ≥99% Ag and ~60 mg of ≥99% CuO. The tubes were evacuated, sealed, and then combusted at 850°C for 3 hours. The resulting CO_2_ was cryogenically purified under vacuum to remove H_2_O and other noncondensable gases and then reduced to graphite via a Bosch reaction in an H_2_ atmosphere at 550°C for 3 hours. During this process, the carbon in CO_2_ is converted to graphite as filaments grown on ≥99% Fe particles, and any remaining H_2_O is removed with magnesium perchlorate during the reaction (*79*). The Fe/C particles were pressed into Al cathodes and loaded into an Al wheel along with process blanks and primary and secondary standards. ^14^C was measured on a National Electronics Corporation compact AMS with a 0.5-MV accelerator (*80*). Analytical error is in the 2 to 3‰ range under beam currents of up to 200 μA of ^12^C*^−^* with routine generation of 120 to 150 μA of ^12^C^−^ from ~0.7-mg C samples. Radiocarbon ages are corrected for mass-dependent fractionation with δ^13^C values measured on the AMS and normalized to OXII standards (SRM-4990C). Median-calibrated radiocarbon dates for each individual were calculated using 10,000 Monte Carlo simulations, weighted by the probability densities of calibrated radiocarbon dates using the IntCal20 calibration curve (*81*), generated in the rcarbon package (*82*) in R.

### Stable isotope mixing models

All data analyses were performed using the statistical programming language R, version 4.3.1 (*83*), within RStudio version 2024.12.0 + 467 (*84*). We used the Bayesian stable isotope mixing model package MixSIAR (*85*); full specifications including priors, MCMC parameters, and error structures are provided in Supplementary Text) to estimate the proportional contributions of essential amino acids from wild and domestic C_3_ plants, C_4_ plants (maize), and C_4_ animal protein (modern maize-fed turkeys as a proxy for ancient maize-eating animals) to the essential amino acids in each individual’s bone collagen. We used a TDF of 0‰ (see Supplementary Text and data S1 for results of models run with ±0.5‰ TDFs), assuming negligible fractionation (direct routing) of essential amino acid δ^13^C values during trophic transfer.

#### Modeling approach

We used a two-step modeling approach to explore different yet complementary research questions regarding dietary contributions. All results are presented as means and SDs derived from computed probability distributions. First, we conducted a single mixing model using the mean δ^13^C values of five essential amino acids (lysine, isoleucine, leucine, phenylalanine, and valine) from C_3_ plants (local native wild and domestic), and C_4_ plants (local varietals of maize). This model provided an overarching estimate of the total contribution of C_4_-derived protein to each individual’s bone collagen, which was crucial for establishing a baseline understanding of dietary trends over time and for providing important contextual information to inform our subsequent analyses (Fig. 6).

Second, to quantify the specific contributions of C_4_ sources to each essential amino acid for each individual, we conducted separate mixing models for lysine, isoleucine, leucine, phenylalanine, and valine using the mean δ^13^C values from C_3_ and C_4_ plants (table S2). The variability in C_4_ proportions for each amino acid correlates with its relative abundance in maize protein (table S4), influencing trophic magnification through trophic transfers. Consequently, the overall proportion of protein derived from a C_4_ source reflects a weighted average based on amino acid concentrations in maize and the differences in routing versus de novo synthesis of nonessential amino acids. This approach enabled us to quantify the C_4_-derived proportion of each amino acid, determine the quantities of C_4_-based food sources necessary to consume to maintain homeostasis, and highlight the unique role of lysine as the limiting protein-based nutrient in maize-rich diets (Figs. 3 and 6). Modeling amino acids separately revealed nuanced dietary patterns and constraints, particularly concerning lysine availability compared to other essential amino acids. Our results established a foundation for estimating the quantities of maize needed to meet dietary amino acid requirements.

### Dietary implications: C_4_ amino acid requirements

Daily requirements for essential amino acids are dependent on both age and weight (table S3 and data S1). We calculated the daily requirement (milligrams) of each essential amino acid to maintain nitrogen balance using estimated body weights derived from osteologically estimated age categories (fig. S4, Fig. 3, and data S1). These values were combined with the C_4_ contributions estimated from the single-amino acid mixing models to determine the daily intake (milligrams) of essential amino acids from a C_4_ source, assumed to be maize. The calculated values (daily milligrams) of amino acids derived from maize were then used to estimate the quantity of dry maize required to meet daily dietary needs based on the amino acid concentrations in maize

$$R_{\text{maize}}=\left({\mathrm{AA}}_{\mathrm{req}}\right)\times \left(P_{C_{4}}\right)\times \left(C_{\text{AA-maize}}\right)$$ (1)

where *R*_maize_ is the daily quantity (grams) of maize or nixtamalized maize, each individual would need to consume to meet daily age- and weight-specific amino acid requirements (*AA*_req_, milligrams). This calculation accounts for the proportion of each amino acid derived from a C_4_ source ($P_{C_{4}}$) and the concentration of that amino acid in maize or nixtamalized maize (*C*_AA-maize_, milligrams per gram). We then multiplied each value by 3 to estimate the wet weights of each food source, providing a more realistic representation.

### Alternative lysine sources: Nixtamalized maize and C_4_-eating animal meat

On the basis of our results, we focused further calculations specifically on lysine. To explore alternative lysine sources, we extended our calculations to nixtamalized maize, substituting its lysine concentration in Eq. 1 for that of dry maize to estimate the age- and weight-dependent amount required to meet daily lysine needs.

For turkey meat, additional calculations were needed to estimate C_4_-derived lysine content in these free-ranging, maize-fed animals. Using the two-source mixing model for lysine, we estimated that the average C_4_-derived lysine in maize-fed turkeys ranged from 40 to 60%, based on the lysine δ^13^C values of modern free-ranging, maize-fed turkeys from villages near our study sites. These values then allowed us to estimate the daily consumption of turkey meat required to meet each individual’s daily C_4_-derived lysine estimates, assuming both a high (60% C_4_ content) and a low (40% C_4_ content) proportion of C_4_-derived lysine in turkey meat. The 40 to 60% C_4_-derived lysine range reflects modern free-ranging maize-fed animals and serves as a conservative baseline parameter. We do not assume that this range captures the full variability of ancient animal diets; instead, we use it to explore nutritionally constrained scenarios under defined model assumptions.

For individuals with lysine δ^13^C values higher than maize-fed turkeys, additional two-source mixing models were applied to differentiate lysine contributions from direct consumption of C_4_ plants (maize) versus maize-eating animals using data for the modern free-ranging turkeys as a proxy (see Supplementary Text for modeling example). These elevated values suggest that the C_4_-derived lysine intake for these individuals may have originated from a combination of maize-eating animals and direct consumption of maize or nixtamalized maize, assuming ancient maize-fed animals could have had similar dietary habits as these modern proxies. Using the turkey model outputs, we conducted another series of two-source mixing models for these individuals to estimate the milligrams of C_4_-derived lysine obtained from maize and maize-eating animals (with 40 and 60% C_4_ contributions) using average lysine δ^13^C values of the two sources (Fig. 6). These values informed calculations of the combined quantities of maize or nixtamalized maize and maize-fed animal meat required to meet daily estimates of C_4_-derived lysine needs. Again, we varied the proportion of C_4_-derived lysine in animal meat from 40 to 60%. This detailed framework enabled us to quantify reliance on maize products and maize-eating animal protein in fulfilling dietary lysine requirements across the study population, highlighting the constraints of maize-based diets and the important role of alternative sources of lysine derived from maize

$$R_{\text{maize}}=\left({\mathrm{AA}}_{\mathrm{req}}\right)\times \left(P_{C_{4}}\right)\times \left(P_{C_{4}\text{-plant}}\right)\times \left(C_{\text{AA-maize}}\right)$$ (2)

$$R_{\text{animal}}=\left({\mathrm{AA}}_{\mathrm{req}}\right)\times \left(P_{C_{4}}\right)\times \left(P_{C_{4}\text{-animal}}\right)\times \left(C_{\text{AA-turkey}}\right)$$ (3)

$$R_{\text{total}}=R_{\text{maize}}+R_{\text{animal}}$$ (4)

where *R*_maize_ and *R*_animal_ represent the daily quantities (grams) of maize or nixtamalized maize and maize-eating animal protein, respectively, that an individual would need to consume to meet their age- and weight-specific amino acid requirements (*AA*_req_, milligrams). These requirements are calculated after accounting for the proportion of each amino acid derived from a C_4_ source ($P_{C_{4}}$), with further differentiation between the fraction contributed by C_4_ plants ($P_{C_{4}}$_-plant_) and that derived from maize-eating animals ($P_{C_{4}}$_-animal_). The final intake requirement is determined by the concentration of the target amino acid (in this case, lysine) in maize (CAA-maize, milligrams per grams) and in maize-eating turkey (C_AA-turkey_, milligrams per gram). The total food requirement (*R*_total_, grams) is met through the combined intake of maize or nixtamalized maize and animal-derived protein sources.

## Supplementary Material

20260708-1

## Notes

**Correction (13 October 2026):** In the original Fig. 1, several values included in the “Turkeys” panel were inadvertently replaced by values from the “Humans” panel. Fig. 1 has been replaced, and the updated values are as follows: “1 g nixtamal” is now “1 g maize”; “816.5mg lysine” is now “157.3 mg lysine”; “3240 g nixtamal” is now “1165 g maize”; “3.2 kg of nixtamal a day” is now “1.2 kg of maize a day”; and “1.4 kg of nixtamal a day” is now “0.6 kg of nixtamal a day.” Both the PDF and online versions of the article have been corrected.

## Supplementary Materials

### The PDF file includes:

Supplementary Text Figs. S1 to S4 Tables S1 to S4 Legend for data S1 Legends for codes S1 to S3 References

### Other Supplementary Material for this manuscript includes the following:

Data S1

Codes S1 to S3
