# Supplementary material for: Nutritional adaptations to early maize cultivation: Earliest isotopic evidence of maize-based animal provisioning in the Neotropics
Source: Sci Adv. 2026 Jul 8;12(28):eaec3522. doi: 10.1126/sciadv.aec3522 (PMC13344295; doi:10.1126/sciadv.aec3522)
Supplement: Supplementary file 1 — Supplementary Text Figs. S1 to S4 Tables S1 to S4 Legend for data S1 Legends for codes S1 to S3 References [file sciadv.aec3522_sm.pdf]

Science Advances
AAAS

Supplementary Materials for

Nutritional adaptations to early maize cultivation: Earliest isotopic evidence of maize-based animal provisioning in the Neotropics

Nadia C. Neff *et al.*

Corresponding author: Nadia C. Neff, ncneff@unm.edu

Sci. Adv. **12**, eaec3522 (2026)
DOI: 10.1126/sciadv.aec3522

The PDF file includes:

Supplementary Text
Figs. S1 to S4
Tables S1 to S4
Legend for data S1
Legends for codes S1 to S3
References

Other Supplementary Material for this manuscript includes the following:

Data S1
Codes S1 to S3

Supplementary Text

Provenance

The human skeletal remains analyzed in this study were originally recovered during archaeological investigations in Belize between 1995 and 2023. Two individuals were excavated from Bats'ub Cave in 1995-1996 as part of the Maya Mountains Archaeological Project. Eight individuals were excavated from Uxbenká and Ix Ku'ku'il by project bioarchaeologists on the Uxbenká Archaeological Project (2004-2015), and the remaining 29 individuals were excavated from Mayahak Cab Pek and Saki Tzul by project bioarchaeologists on the Research into the Origins and Organization of Tropical Societies (ROOTS) project (2014-2026). All excavations were conducted under permits issued to KMP by the Belize Institute of Archaeology, the National Institute of Culture and History (IA-NICH), and the Belize National Biodiversity Office. All samples were exported to the USA under permits from the Belize Institute of Archaeology, with specific permission to conduct molecular analyses. Archaeological provenience information was recorded during excavation in accordance with established field protocols, and these records are curated at the University of New Mexico. The authenticity and archaeological context of the human remains were evaluated by trained bioarchaeologists and collaborating specialists through osteological and stratigraphic observations, associated material culture, and comparison with previously documented site archaeological sequences. The chronology was established through direct AMS radiocarbon dating of human remains, as described in the Materials and Methods section. All archaeological samples remain the property of the people and Government of Belize and are curated under permit at the University of New Mexico Maxwell Museum of Anthropology. Any researchers seeking to conduct research on these materials would be required to request permission from the project PI, KMP, and the Belize Institute of Archaeology. All data generated as part of this study are included in the main text and/or the supplementary materials.

Study Area

The area of study for this paper is located in present-day southern Belize, part of the northern extent of the neotropics, a region characterized by mountainous tropical and subtropical landscapes that receive over 4,000mm of rain/year (*86, 87*). This region of the Southern Maya Lowlands is dominated by the volcanic Maya Mountains, carbonate platforms, swampy coastal plains, and colluvial savannas (*88, 89*). Archaeological samples were collected from the sites of Mayahak Cab Pek (MHCP), Saki Tzul (ST), Uxbenká (UXB), or Ix Kuku'il (IK), and modern samples were gathered from locations near these archaeological sites.

Mayahak Cab Pek and Saki Tzul are rockshelters located in the Ek Xux Valley of the Bladen Nature Reserve (*90*). They were not sites of permanent human habitation but were instead used for interring the dead, food processing, and religious activities (*91*). Radiocarbon dating indicates these sites were in use from ~12,500 BP to ~1,000 BP, with evidence of nearly continuous archaeological depositions of human and faunal remains over a span of 10,000 years (*2*). Recent excavations conducted from 2014 to 2024 have yielded a combined skeletal minimum number of individuals (MNI) of 65. The rockshelters are notable for their exceptional preservation of neotropical skeletal remains and include some of the earliest Archaic and Paleoindian human remains in Mesoamerica.

Uxbenká and Ix Kuku'il are medium-sized polities located in the foothills of the southern Maya

Mountains in the Toledo district of southern Belize. Along with other sites in the foothills, these polities are situated on the fertile soils of the Toledo Uplands known for their high agricultural productivity (*92*). The site cores and surrounding settlements have been extensively surveyed and excavated, resulting in a robust chronology based on AMS radiocarbon dating (*93*). Together, these sites have a combined skeletal MNI of 53 (Uxbenká, n = 42, Ix Kuku'il, n = 11). Both sites were continuously occupied for over 10 centuries, from the Late Preclassic until their abandonment after 1,000 BP (*94*).

Today, this region is predominantly covered by broadleaf forests, which include significant food and agroforestry crops. The seasonal rainfall sustains an evergreen rainforest characterized by a dense and tall canopy (40-70m), allowing little sunlight to reach the forest floor without human intervention. These closed-canopy rainforests are interspersed with coniferous forests and mangrove trees, palmetto savannas, and anthropogenically modified landscapes (*53, 54*). Nearly all native wild and domesticated plants in this region use a C_3 photosynthetic pathway, except for a few low-abundance C_4 grasses found in nearby savannahs.

Stable Isotope Mixing Models

To estimate the proportional contributions of C_3 plants (wild and domestic), C_4 plants (maize), and maize-fed turkeys to each individual's essential amino acids, we used the Bayesian stable isotope mixing model framework, MixSIAR (*85*) which is available as an open-source package in R. For each model, we assumed a trophic discrimination factor of 0‰, as only one or more essential amino acids were included in the models, and theoretically there is no fractionation of essential amino acid carbon through trophic exchanges.

Markov chain Monte Carlo parameters were set to the following: "long" run length: chain length = 300,000, burn = 200,000, thin = 500, chains = 3; Dirichlet prior: = 1, Dirichlet prior (α = 1, uninformative); residual error = FALSE, process error = TRUE. This error structure is appropriate for models fit to single consumers rather than a group, as there is no information about within-consumer variability, making a residual error unsuitable (*85*).

We conducted a series of MixSIAR models for each individual, starting with a multivariate two-source model that used mean $\delta^{13}C$ values from C_3 (wild and domestic plants) and C_4 (maize) of five essential amino acids measured (lysine, isoleucine, leucine, phenylalanine, and valine). Subsequently, we ran separate models for each essential amino acid to determine the proportion of each derived from C_3 versus C_4 sources. All results are reported as means and standard deviations based on the computed probability distributions.

Modeling Example

As a methodological example, individual MHCP.14.1.3 has a lysine $\delta^{13}C$ value of -15.3‰ (Data S1). Assuming an average weight of 68kg, this individual required 816mg of lysine daily. 48.2% of their lysine intake was derived from a C_4 source (MixSIAR model output 1: C_3 vs. C_4), with 393mg (48.2%) of their total 816mg of lysine derived from C_4 sources. Of this, 335mg (85.3%) came from C_4 fauna and 58mg (14.7%) from C_4 plants (MixSIAR model output 2: C_4 fauna vs. C_4 plants), translating to 39g of turkey meat (40% C_4 content) or 26g (60% C_4 content) combined with 430g of dry maize or 230g of dry nixtamalized maize (Fig. 5a). These estimates suggest a

significant reliance on both C_4 plants and animals, with the possibility of higher or lower C_4-derived lysine in ancient C_4-consuming animals than observed in these modern proxies.

Sensitivity Analyses

To evaluate the robustness of our modeled C_4-derived proportions and the resulting dietary requirement estimates for maize and nixtamalized maize, we conducted a series of sensitivity analyses with different key physiological and isotopic parameters. First, we examined the effects of small yet significant potential trophic discrimination factors (TDFs) on $\delta^{13}C$ values of essential amino acids in our MixSIAR models. Although essential amino acids are generally assumed to be routed into consumer tissues with negligible fractionation, we reran all lysine-based mixing models using TDFs of +0.5 ‰ and -0.5 ‰, in addition to the baseline assumption of 0 ‰. These alternative models, therefore, produced three sets of posterior mean (±SD) C_4-derived lysine proportions for each individual. Second, we assessed potential uncertainty in some key physiological assumptions by varying estimated body weights (±20 %) and daily lysine requirements (mg/kg/day; ±20 %) for each individual, based on average body weights by age group. These values, along with the three models with different TDFs, were then incorporated into 27 recalculations of daily lysine requirements and the corresponding amounts of maize, nixtamalized maize, and maize-eating animal protein.

All 27 possible parameter combinations (TDF variation x body weight variation x lysine requirement variation) were calculated programmatically in R (see accompanying .Rmd files). The summary outputs include the mean, minimum, and maximum dietary requirement estimates for each individual. These results are provided in expanded columns within Data S1, tab: Human Model Results, and the full combination outputs are included in the tab: Sensitivity Analyses. Across all tested parameter combinations, the qualitative interpretation of the models remained unchanged when contextualized with archaeological data on maize size, and kernel and seed row counts of early maize. Even under conservative assumptions that minimized C_4-derived lysine requirements, the direct consumption of maize or nixtamalized maize alone would require sustained intake levels that approach or exceed realistic daily dietary intakes, especially given the need to obtain the remaining lysine from C_3-based foods.

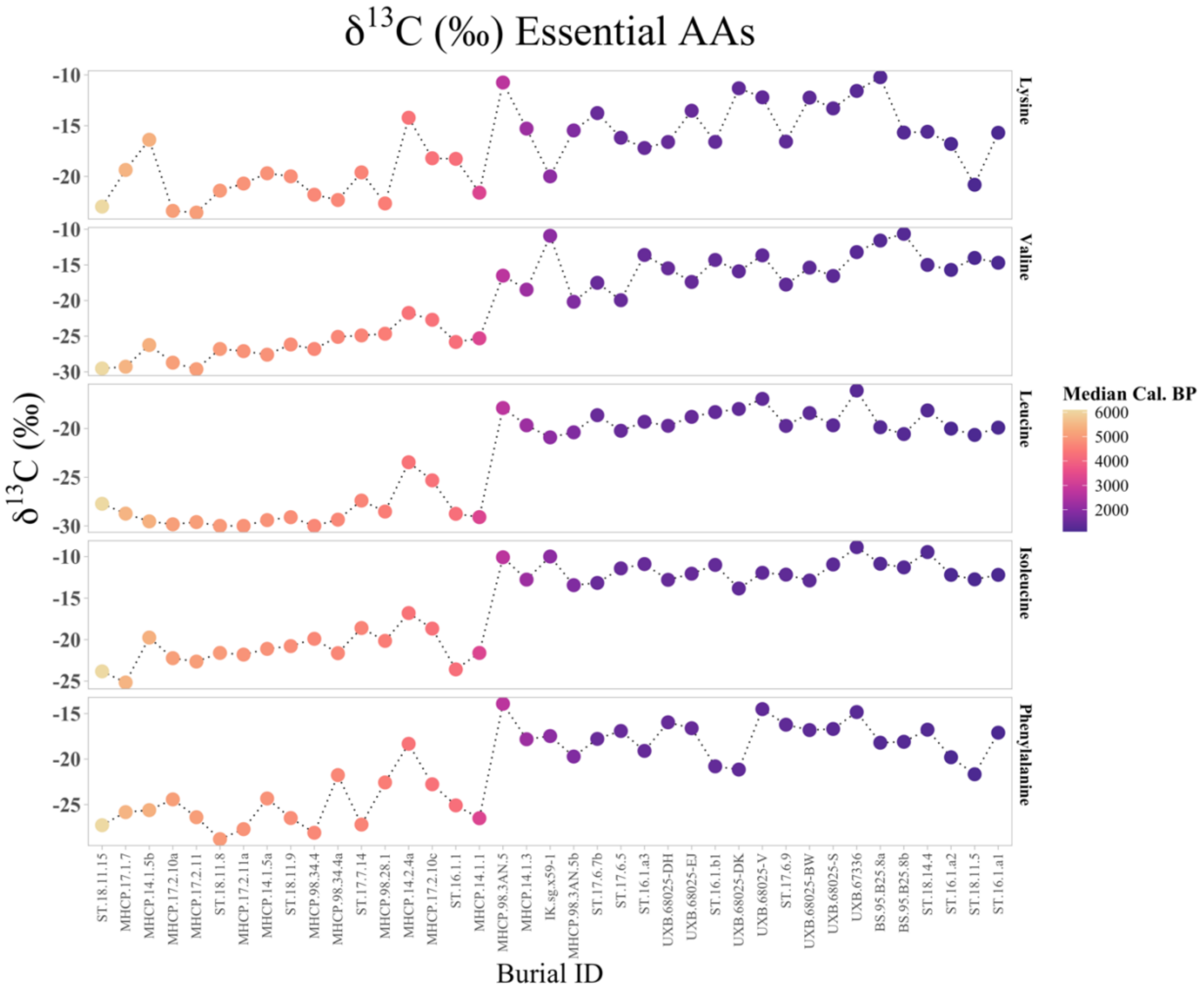

Fig. S1. δ¹³C values for essential amino acids (EAAs) over time. Measured δ¹³C values (‰) for five essential amino acids, lysine, valine, leucine, isoleucine, and phenylalanine, are shown across individual burials. Burial IDs are arranged chronologically (left to right), and color indicates median calibrated radiocarbon age (Cal. BP). A clear temporal shift toward elevated δ¹³C values is observed across all EAAs, consistent with increasing dietary C_4 input through time.

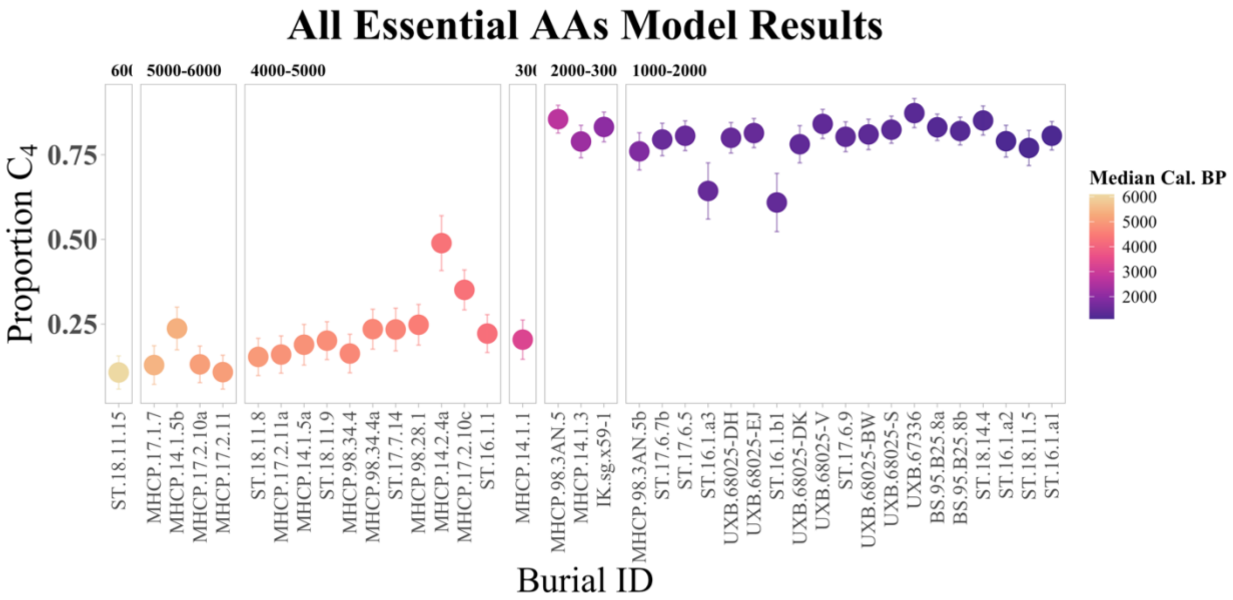

Fig. S2. Proportion of C_4-derived EAAs from the all-EAA model. Estimated dietary C_4 contribution derived from Bayesian modeling of all five essential amino acids combined. Error bars represent 95% credible intervals. Burial IDs are grouped by temporal bins and colored by median Cal. BP. These results illustrate a substantial increase in C_4-derived protein starting after ~4000 BP.

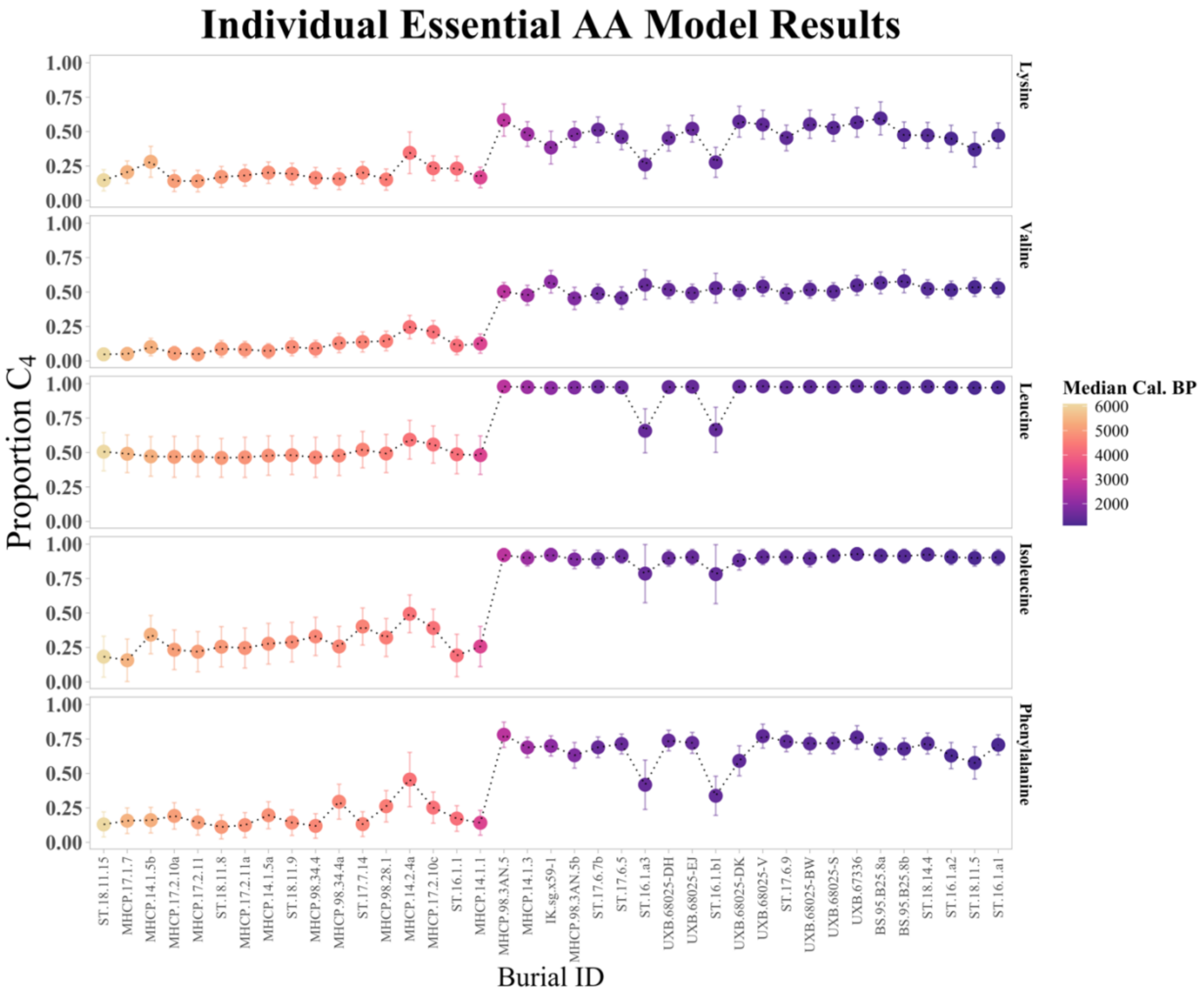

Fig. S3. Proportion of C_4 contribution to individual EAAs. Modeled proportion of C_4-derived lysine, valine, leucine, isoleucine, and phenylalanine for each burial. Error bars represent 95% credible intervals. Burials are arranged chronologically and colored by median Cal. BP. The temporal coherence across amino acids supports increasing reliance on maize and other C_4 resources over time.

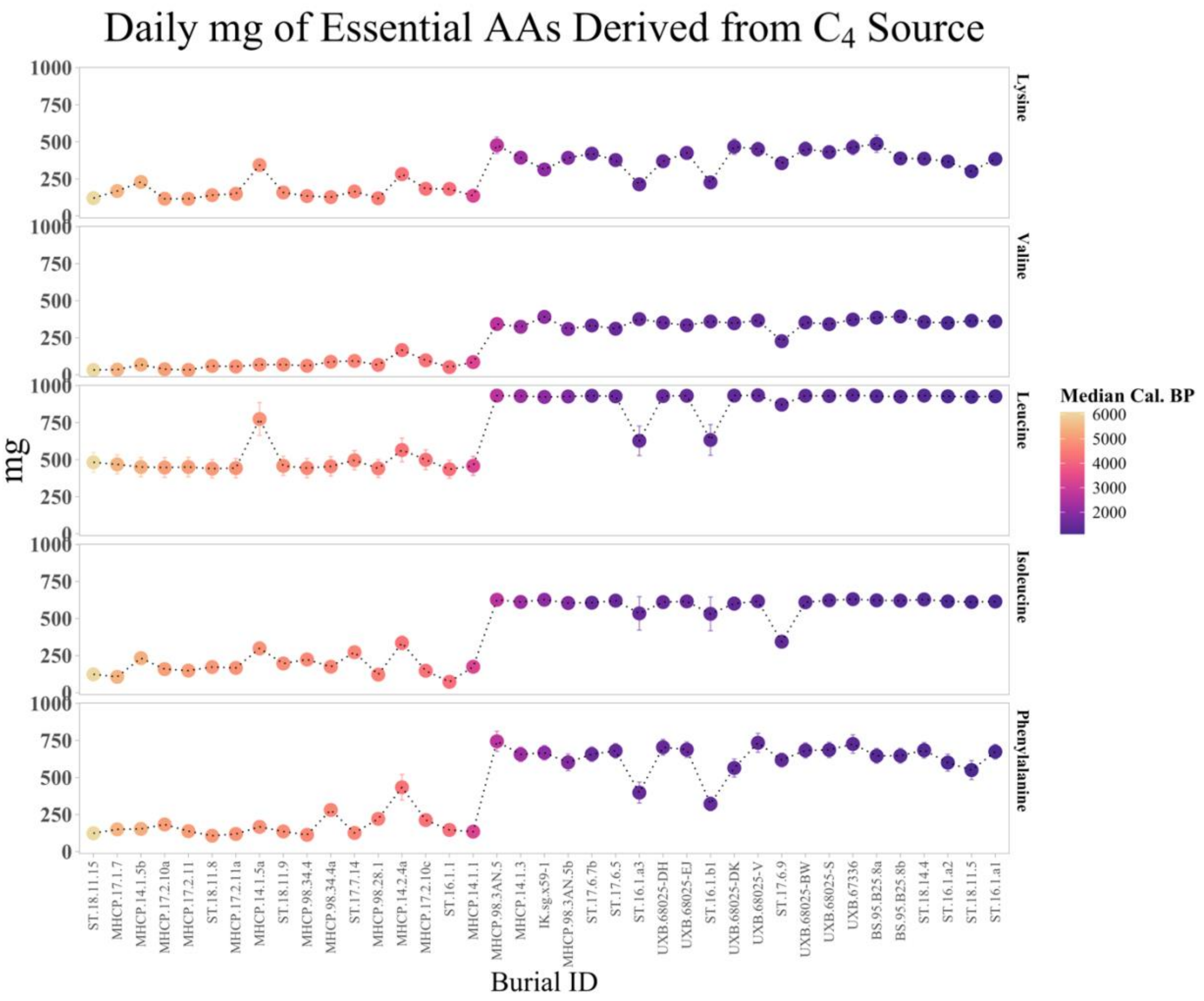

Fig. S4. Daily intake of essential AAs derived from C_4 sources. Estimated daily milligram intake of each essential amino acid attributable to C_4 sources, based on isotopic mixing models and published dietary requirements. Colored points reflect median Cal. BP. Results indicate that C_4 sources eventually supplied the majority of daily essential amino acids, key metrics for evaluating nutritional adequacy in maize-based diets.

Table S1.

Amino Acid	Proportion of C added during derivatization	In-house reference material know $\delta^{13}C$ values	Mean within-run $\delta^{13}C$ SD
**Valine**	0.5	-11.8	0.2
**Leucine**	0.45	-28.3	0.3
**Isoleucine**	0.45	-12.1	0.2
**Phenylalanine**	0.36	-13.1	0.3
**Lysine**	0.54	-19.9	0.4

In-house reference material $\delta^{13}C$ values with mean within-run standard deviations.

Table S2.

Amino Acid	C_3 $\delta^{13}C$		C_4 $\delta^{13}C$	
	**Mean**	**SD**	**Mean**	**SD**
Lysine	-23.54	4.95	-5.47	3.95
Leucine	-35.03	3.3	-19.79	2.4
Isoleucine	-26.05	4.18	-10.08	2.27
Phenylalanine	-27.62	3.47	-12.87	1.37
Valine	-29.5	5.01	-2.09	6.45
**n**	**38**	**38**	**10**	**10**

Mean values for five essential amino acids used in mixing models as source groups. These values were derived from a sample of 48 modern plants collected in the study region over several years. These plants included wild and domesticated C_3 plants and domesticated C_4 plants.

Table S3.

Amino Acid	mg/kg bodyweight per day				Daily requirements (mg) for average weight			
	3-4 months	2 years	10-12 years	Adults	3-4 months	2 years	10-12 Years	Adults
Isoleucine	70	31	28	10	460.4	379.7	1079.5	680.4
Leucine	161	73	42	14	1058.9	894.0	1619.3	952.5
Lysine	103	64	44	12	677.4	783.8	1696.4	816.5
Phenylalanine	125	69	22	14	822.1	845.0	848.2	952.5
Valine	93	38	25	10	611.7	465.4	963.9	680.4

Daily essential amino acid requirements by age and weight (*38*).

Table S4.

Amino Acid	Turkey Meat		Dry Maize	
	mg/100g	% of protein	mg/100g	% of protein
**Ile**	773	3.39	342	3.8
**Leu**	1869	8.19	1683	18.7
**Lys**	2225	9.75	13.5	0.15
**Phe**	869	3.81	468	5.2
**Val**	862	3.78	324	3.6

Essential amino acid composition of turkey meat and dry maize.

Data S1. (separate file)
This file includes four tabs. Tab 1 presents data for each individual in this study: bulk and compound-specific isotopic data, radiocarbon data, calculated means and medians, sex, age, age categories, and archaeological site. Tab 2 provides all the results of the mixing models for each individual. Tab 3 shows the turkey stable isotope and mixing model results. Tab 4 reports the results of all 27 parameter combinations for each individual for the model sensitivity analyses.

Code S1. (separate file)
This is an R Markdown file that contains the code to run each mixing model used in this study. It is annotated with instructions for executing specific sets of models based on the amino acids of interest, and it includes a series of three TDFs that can be adjusted for sensitivity analysis. This file corresponds to the Data S1 file and uses the data in this Excel file in its current format to perform the analyses.

Code S2. (separate file)
This is the HTML output from an R Markdown file that contains the code to generate all figures in the main manuscript and the supplemental information, along with the figures themselves.

Code S3. (separate file)
This is an R Markdown file that contains extra code for a series of sensitivity analyses requested by a reviewer. It corresponds to the Data S1 file and uses the data in this Excel file in its current format to perform the analyses.

REFERENCES

1. C. S. Larsen, The agricultural revolution as environmental catastrophe: Implications for health and lifestyle in the Holocene. *Quat. Int.* **150**, 12–20 (2006).

2. D. J. Kennett, K. M. Prufer, B. J. Culleton, R. J. George, M. Robinson, W. R. Trask, G. M. Buckley, E. Moes, E. J. Kate, T. K. Harper, L. O'Donnell, E. E. Ray, E. C. Hill, A. Alsgaard, C. Merriman, C. Meredith, H. J. Edgar, J. J. Awe, S. M. Gutierrez, Early isotopic evidence for maize as a staple grain in the Americas. *Sci. Adv.* **6**, eaba3245 (2020).

3. P. Bellwood, *First Farmers: The Origins of Agricultural Societies* (Wiley, 2023).

4. L. J. Reitsema, Beyond diet reconstruction: Stable isotope applications to human physiology, health, and nutrition. *Am. J. Hum. Biol.* **25**, 445–456 (2013).

5. V. R. Young, P. L. Pellett, Plant proteins in relation to human protein and amino acid nutrition. *Am. J. Clin. Nutr.* **59**, 1203S–1212S (1994).

6. J. A. Villada, F. Sanchez-Sinencio, O. Zelaya-Angel, E. Gutierrez-Cortez, M. E. Rodríguez-García, Study of the morphological, structural, thermal, and pasting corn transformation during the traditional nixtamalization process: From corn to tortilla. *J. Food Eng.* **212**, 242–251 (2017).

7. D. Zizumbo-Villarreal, A. Flores-Silva, P. Colunga-García Marín, The archaic diet in Mesoamerica: Incentive for milpa development and species domestication. *Econ. Bot.* **66**, 328–343 (2012).

8. A. E. Sharpe, K. F. Emery, T. Inomata, D. Triadan, G. D. Kamenov, J. Krigbaum, Earliest isotopic evidence in the Maya region for animal management and long-distance trade at the site of Ceibal, Guatemala. *Proc. Natl. Acad. Sci. U.S.A.* **115**, 3605–3610 (2018).

9. M. A. Zeder, D. G. Bradley, B. D. Smith, E. Emshwiller, "Documenting domestication: brining together plants, animals, archaeology, and genetics," in *Documenting Domestication: New Genetic and Archaeological Paradigms,* M. A. Zeder, Ed. (University of California Press, Oakland, CA, 2006).

10. E. K. Thornton, K. F. Emery, D. W. Steadman, C. Speller, R. Matheny, D. Yang, Earliest Mexican turkeys (Meleagris gallopavo) in the Maya region: Implications for pre-Hispanic animal trade and the timing of turkey domestication. *PLOS ONE* **7**, e4230 (2012).

11. P. L. Walker, R. R. Bathurst, R. Richman, T. Gjerdrum, V. A. Andrushko, The causes of porotic hyperostosis and cribra orbitalia: A reappraisal of the iron-deficiency-anemia hypothesis. *Am. J. Phys. Anthropol.* **139**, 109–125 (2009).

12. Y. Matsuoka, Y. Vigouroux, M. M. Goodman, J. Sanchez G, E. Buckler, J. Doebley, A single domestication for maize shown by multilocus microsatellite genotyping. *Proc. Natl. Acad. Sci. U.S.A.* **99**, 6080–6084 (2002).

13. L. Chen, J. Luo, M. Jin, N. Yang, X. Liu, Y. Peng, W. Li, A. Phillips, B. Cameron, J. S. Bernal, Genome sequencing reveals evidence of adaptive variation in the genus Zea. *Nat. Genet.* **54**, 1736–1745 (2022).

14. D. R. Piperno, The origins of plant cultivation and domestication in the New World tropics: Patterns, process, and new developments. *Curr. Anthropol.* **52**, S453–S470 (2011).

15. L. Kistler, S. Y. Maezumi, J. Gregorio de Souza, N. A. Przelomska, F. Malaquias Costa, O. Smith, H. Loiselle, J. Ramos-Madrigal, N. Wales, E. R. Ribeiro, Multiproxy evidence highlights a complex evolutionary legacy of maize in South America. *Science* **362**, 1309–1313 (2018).

16. W. L. Merrill, R. J. Hard, J. B. Mabry, G. J. Fritz, K. R. Adams, J. R. Roney, A. C. MacWilliams, The diffusion of maize to the southwestern United States and its impact. *Proc. Natl. Acad. Sci. U.S.A.* **106**, 21019–21026 (2009).

17. J. Ramos-Madrigal, G. J. Fritz, B. Schroeder, B. Smith, F. Sánchez-Barreiro, C. Carøe, A. K. W. Runge, S. Boer, K. McGrath, F. G. Vieira, The genomic origin of early maize in eastern North America. *Cell* **188**, 33–43.e16 (2025).

18. K. O. Pope, M. E. Pohl, J. G. Jones, D. L. Lentz, C. v. Nagy, F. J. Vega, I. R. Quitmyer, Origin and environmental setting of ancient agriculture in the lowlands of Mesoamerica. *Science* **292**, 1370–1373 (2001).

19. B. N. Smith, S. Epstein, Two categories of $^{13}C/^{12}C$ ratios for higher plants. *Plant Physiol.* **47**, 380–384 (1971).

20. E. E. Ray, N. C. Neff, P. Lynch, J. Mes, M. S. Lachniet, D. J. Kennett, K. M. Prufer, The development of early farming diets and population change in the Maya region and their climate context. *Quat. Int.* **689**, 6–78 (2024).

21. T. Larsen, M. Ventura, N. Andersen, D. M. O'Brien, U. Piatkowski, M. D. McCarthy, Tracing carbon sources through aquatic and terrestrial food webs using amino acid stable isotope fingerprinting. *PLOS ONE* **8**, e73441 (2013).

22. B. Finucane, P. M. Agurto, W. H. Isbell, Human and animal diet at Conchopata, Peru: Stable isotope evidence for maize agriculture and animal management practices during the Middle Horizon. *J. Archaeol. Sci.* **33**, 1766–1776 (2006).

23. M. J. LeFebvre, S. D. deFrance, G. D. Kamenov, W. F. Keegan, J. Krigbaum, The zooarchaeology and isotopic ecology of the Bahamian hutia (Geocapromys ingrahami): Evidence for pre-Columbian anthropogenic management. *PLOS ONE* **14**, e0220284 (2019).

24. A. D. Somerville, N. Sugiyama, L. R. Manzanilla, M. J. Schoeninger, Animal management at the ancient metropolis of Teotihuacan, Mexico: Stable isotope analysis of leporid (cottontail and jackrabbit) bone mineral. *PLOS ONE* **11**, e0159982 (2016).

25. J. M. Capriles, C. M. Santoro, R. J. George, E. Flores Bedregal, D. J. Kennett, L. Kistler, F. Rothhammer, Pre-Columbian transregional captive rearing of Amazonian parrots in the Atacama Desert. *Proc. Natl. Acad. Sci. U.S.A.* **118**, e2020020118 (2021).

26. A. D. Somerville, B. A. Nelson, K. J. Knudson, Isotopic investigation of pre-Hispanic macaw breeding in Northwest Mexico. *J. Anthropol. Archaeol.* **29**, 125–135 (2010).

27. N. Sugiyama, A. D. Somerville, M. J. Schoeninger, Stable isotopes and zooarchaeology at Teotihuacan, Mexico reveal earliest evidence of wild carnivore management in Mesoamerica. *PLOS ONE* **10**, e0135635 (2015).

28. N. Sugiyama, M. F. Martínez-Polanco, C. A. France, R. G. Cooke, Domesticated landscapes of the neotropics: Isotope signatures of human-animal relationships in pre-Columbian Panama. *J. Anthropol. Archaeol.* **59**, 101195 (2020).

29. E. Thornton, K. F. Emery, C. Speller, Ancient Maya turkey husbandry: Testing theories through stable isotope analysis. *J. Archaeol. Sci. Rep.* **10**, 584–595 (2016).

30. E. K. Thornton, K. F. Emery, The uncertain origins of Mesoamerican turkey domestication. *J. Archaeol. Method Theory* **24**, 328–351 (2017).

31. A. D. Somerville, M. Fauvelle, A. W. Froehle, Applying new approaches to modeling diet and status: Isotopic evidence for commoner resiliency and elite variability in the Classic Maya lowlands. *J. Archaeol. Sci.* **40**, 1539–1553 (2013).

32. M. A. Masson, C. P. Lope, "Animal consumption at the monumental center of Mayapán," in *The Archaeology of Mesoamerican Animals*, C. M. Götz, K. F. Emery, Eds. (Lockwood Press, 2013), pp. 233–279.

33. C. D. White, M. D. Pohl, H. P. Schwarcz, F. J. Longstaffe, "Deer and Dog Diets at Lagartero, Tikal, and Copán," in *Maya zooarchaeology: New Directions in Method and Theory*, K. F. Emery, Ed. (Cotsen Institute of Archaeology Press, 2004), vol. 51, pp. 141–158.

34. C. D. White, Stable isotopes and the human-animal interface in Maya biosocial and environmental systems. *Archaeofauna* **13**, 183–198 (2004).

35. M. Hashim, I. Alam, M. Ahmad, Badruddeen, J. Akhtar, M. I. Khan, A. Islam, S. Parveen, Comprehensive review of L-lysine: Chemistry, occurrence, and physiological roles. *Curr. Protein Pept. Sci.* **26**, PMID40626529 (2025).

36. Food and Agriculture Organization of the United Nations, *Maize in Human Nutrition* (FAO Food and Nutrition Series, FAO, Rome, 1992), vol. 25, p. 168.

37. Y. Ai, J. l. Jane, Macronutrients in corn and human nutrition. *Compr. Rev. Food Sci. Food Saf.* **15**, 581–598 (2016).

38. National Research Council (US) Subcommittee on the Tenth Edition of the Recommended Dietary Allowances, “Protein and Amino Acids,” in *Recommended Dietary Allowances: 10th Edition* (National Academies Press, ed. 10, 1989), p. 25144070.

39. R. Bressani, R. Paz y Paz, N. S. Scrimshaw, Corn nutrient losses, chemical changes in corn during preparation of tortillas. *J. Agric. Food Chem.* **6**, 770–774 (1958).

40. R. Bressani, Chemistry, technology, and nutritive value of maize tortillas. *Food Rev. Int.* **6**, 225–264 (1990).

41. C. f. F. S. a. P. Health, in *Foreign Animal Disease Prepardness and Response Plan*, D. o. Agriculture, Ed. (Iowa State University, 2013).

42. J. P. Whiteman, E. A. Elliott Smith, A. C. Besser, S. D. Newsome, A guide to using compound-specific stable isotope analysis to study the fates of molecules in organisms and ecosystems. *Diversity* **11**, 8 (2019).

43. T. O’Connell, ‘Trophic’ and ‘source’ amino acids in trophic estimation: A likely metabolic explanation. *Oecologia* **184**, 317–326 (2017).

44. S. H. Payne, W. F. Loomis, Retention and loss of amino acid biosynthetic pathways based on analysis of whole-genome sequences. *Eukaryot. Cell* **5**, 272–276 (2006).

45. G. Wu, Amino acids: Metabolism, functions, and nutrition. *Amino Acids* **37**, 1–17 (2009).

46. S. Jim, V. Jones, S. H. Ambrose, R. P. Evershed, Quantifying dietary macronutrient sources of carbon for bone collagen biosynthesis using natural abundance stable carbon isotope analysis. *Br. J. Nutr.* **95**, 1055–1062 (2006).

47. G. Connolly, J. L. Hudson, R. E. Bergia, E. M. Davis, A. S. Hartman, W. Zhu, C. C. Carroll, W. W. Campbell, Effects of consuming ounce-equivalent portions of animal-vs. plant-based protein foods, as defined by the dietary guidelines for Americans on Essential Amino Acids

Bioavailability in Young and Older Adults: Two cross-over randomized controlled trials. *Nutrients* **15**, 2870 (2023).

48. I. Berrazaga, V. Micard, M. Gueugneau, S. Walrand, The role of the anabolic properties of plant-versus animal-based protein sources in supporting muscle mass maintenance: A critical review. *Nutrients* **11**, 1825 (2019).

49. G. S. Gilani, C. W. Xiao, K. A. Cockell, Impact of antinutritional factors in food proteins on the digestibility of protein and the bioavailability of amino acids and on protein quality. *Br. J. Nutr.* **108**, S315–S332 (2012).

50. S. Jim, S. H. Ambrose, R. P. Evershed, Stable carbon isotopic evidence for differences in the dietary origin of bone cholesterol, collagen and apatite: Implications for their use in palaeodietary reconstruction. *Geochim. Cosmochim. Acta* **68**, 61–72 (2004).

51. R. J. Reid-McCann, S. F. Brennan, M. C. McKinley, C. T. McEvoy, The effect of animal versus plant protein on muscle mass, muscle strength, physical performance and sarcopenia in adults: Protocol for a systematic review. *Syst. Rev.* **11**, 64 (2022).

52. L. Rey, Y. I. Naito, Y. Chikaraishi, S. Rottier, G. Goude, N. Ohkouchi, Specifying subsistence strategies of early farmers: New results from compound-specific isotopic analysis of amino acids. *Int. J. Osteoarchaeol.* **32**, 654–668 (2022).

53. N. Dunning, T. Beach, P. Farrell, S. Luzzadder-Beach, Prehispanic agrosystems and adaptive regions in the Maya lowlands. *Cult. Agric.* **20**, 87–101 (1998).

54. S. L. Fedick, "An Interpretive Kaleidoscope: Alternative Perspectives on Ancient Agricultural Landscapes of the Maya Lowlands," in *The Managed Mosaic: Ancient Maya Agriculture and Resource Use*, S. L. Fedick, Ed. (University of Utah Press, 1996).

55. J. Dombrosky, A~ 1000-year 13C Suess correction model for the study of past ecosystems. *Holocene* **30**, 474–478 (2020).

56. E. J. Guiry, P. Szpak, Improved quality control criteria for stable carbon and nitrogen isotope measurements of ancient bone collagen. *J. Archaeol. Sci.* **132**, 105416 (2021).

57. P. Vaiglova, N. A. Lazar, E. A. Stroud, E. Loftus, C. A. Makarewicz, Best practices for selecting samples, analyzing data, and publishing results in isotope archaeology. *Quat. Int.* **650**, 86–100 (2023).

58. A. Manin, E. Corona-M, M. Alexander, A. Craig, E. K. Thornton, D. Y. Yang, M. Richards, C. F. Speller, Diversity of management strategies in Mesoamerican turkeys: Archaeological, isotopic and genetic evidence. *R. Soc. Open Sci.* **5**, 171613 (2018).

59. D. Wahl, R. Byrne, T. Schreiner, R. Hansen, Holocene vegetation change in the northern Peten and its implications for Maya prehistory. *Quatern. Res.* **65**, 380–389 (2006).

60. S. Morell-Hart, L. Dussol, S. L. Fedick, Agriculture in the ancient Maya lowlands (Part 1): Paleoethnobotanical residues and new perspectives on plant management. *J. Archaeol. Res.* **31**, 561–615 (2023).

61. D. J. Kennett, H. B. Thakar, A. M. VanDerwarker, D. L. Webster, B. J. Culleton, T. K. Harper, L. Kistler, T. E. Scheffler, K. Hirth, High-precision chronology for Central American maize diversification from El Gigante rockshelter, Honduras. *Proc. Natl. Acad. Sci. U.S.A.* **114**, 9026–9031 (2017).

62. J. M. Broughton, E. M. Weitzel, Population reconstructions for humans and megafauna suggest mixed causes for North American Pleistocene extinctions. *Nat. Commun.* **9**, 5441 (2018).

63. M. A. Masson, C. P. Lope, Animal use at the Postclassic Maya center of Mayapán. *Quat. Int.* **191**, 170–183 (2008).

64. P. W. Stahl, M. C. Muse, D. E. Florencio, New evidence for pre-Columbian Muscovy Duck Cairina moschata from Ecuador. *Ibis* **148**, 657–663 (2006).

65. S. R. Orsini, "From Turkeys to Tamales: Paleoindian to Preclassic Period Faunal Use at Maya Hak Cab Pek Rockshelter in Southern Belize," thesis, University of Mississippi, University, MS, eGrove (2016).

66. L. Dotterweich, in *AFIA Feed Bites*, (American Feed Industry Association, Arlington, VA, 2019), vol. 2024.

67. M. Broadbent, H. Bean, The yield of edible meat from turkeys, ducklings, and different market classes of chickens. *Poult. Sci.* **31**, 447–450 (1952).

68. N. Yang, Y. Wang, X. Liu, M. Jin, M. Vallebueno-Estrada, E. Calfee, L. Chen, B. P. Dilkes, S. Gui, X. Fan, Two teosintes made modern maize. *Science* **382**, eadg8940 (2023).

69. S. D. Newsome, K. L. Feeser, C. J. Bradley, C. Wolf, C. Takacs-Vesbach, M. L. Fogel, Isotopic and genetic methods reveal the role of the gut microbiome in mammalian host essential amino acid metabolism. *Proc. R. Soc. B* **287**, 20192995 (2020).

70. C. Cagnato, Gathering and sowing across the central maya lowlands: A review of plant use by preceramic peoples and the early to middle preclassic maya. *Ancient Mesoam.* **32**, 486–501 (2021).

71. S. L. Fedick, Maya cornucopia: Indigenous food plants of the Maya Lowlands. *The Real Business of Ancient Maya Economies: From Farmers' Fields to Rulers'*, (Gainesville, FL, 2020), pp. 224–237.

72. R. Bressani, A. Sánchez-Marroquín, E. Morales, Chemical composition of grain amaranth cultivars and effects of processing on their nutritional quality. *Food Rev. Int.* **8**, 23–49 (1992).

73. D. J. Kennett, M. Lipson, K. M. Prufer, D. Mora-Marín, R. J. George, N. Rohland, M. Robinson, W. R. Trask, H. H. Edgar, E. C. Hill, South-to-north migration preceded the advent of intensive farming in the Maya region. *Nat. Commun.* **13**, 1530 (2022).

74. R. Longin, New method of collagen extraction for radiocarbon dating. *Nature* **230**, 241–242 (1971).

75. A. C. Besser, E. A. Elliott Smith, S. D. Newsome, Assessing the potential of amino acid δ13C and δ15N analysis in terrestrial and freshwater ecosystems. *J. Ecol.* **110**, 935–950 (2022).

76. J. Silfer, M. Engel, S. Macko, E. Jumeau, Stable carbon isotope analysis of amino acid enantiomers by conventional isotope ratio mass spectrometry and combined gas chromatography/isotope ratio mass spectrometry. *Anal. Chem.* **63**, 370–374 (1991).

77. A. L. Robinson, E. A. Elliott Smith, A. C. Besser, S. D. Newsome, Tissue-specific carbon isotope patterns of amino acids in southern sea otters. *Oecologia* **204**, 13–24 (2024).

78. E. A. Elliott Smith, M. T. Tinker, E. L. Whistler, D. J. Kennett, R. L. Vellanoweth, D. Gifford-Gonzalez, M. G. Hylkema, S. D. Newsome, Reductions in the dietary niche of southern sea otters (Enhydra lutris nereis) from the Holocene to the Anthropocene. *Ecol. Evol.* **10**, 3318–3329 (2020).

79. D. J. Kennett, S. Plog, R. J. George, B. J. Culleton, A. S. Watson, P. Skoglund, N. Rohland, S. Mallick, K. Stewardson, L. Kistler, Archaeogenomic evidence reveals prehistoric matrilineal dynasty. *Nat. Commun.* **8**, 14115 (2017).

80. M. Stuiver, H. A. Polach, Discussion reporting of 14C data. *Radiocarbon* **19**, 355–363 (1977).

81. P. J. Reimer, W. E. Austin, E. Bard, A. Bayliss, P. G. Blackwell, C. B. Ramsey, M. Butzin, H. Cheng, R. L. Edwards, M. Friedrich, The IntCal20 Northern Hemisphere radiocarbon age calibration curve (0–55 cal kBP). *Radiocarbon* **62**, 725–757 (2020).

82. E. R. Crema, A. Bevan, Inference from large sets of radiocarbon dates: Software and methods. *Radiocarbon* **63**, 23–39 (2021).

83. R. C. Team, R: A language and environment for statistical computing. *Foundation for Statistical Computing, Vienna, Austria* (2013); http://R-project.org/.

84. RstudioTeam, RStudio: Integrated Development for R. RStudio, PBC (Boston, MA, 2020); http://rstudio.com.

85. B. C. Stock, A. L. Jackson, E. J. Ward, A. C. Parnell, D. L. Phillips, B. X. Semmens, Analyzing mixing systems using a new generation of Bayesian tracer mixing models. *PeerJ* **6**, e5096 (2018).

86. H. E. Ridley, Y. Asmerom, J. U. Baldini, S. F. Breitenbach, V. V. Aquino, K. M. Prufer, B. J. Culleton, V. Polyak, F. A. Lechleitner, D. J. Kennett, Aerosol forcing of the position of the intertropical convergence zone since AD 1550. *Nat. Geosci.* **8**, 195–200 (2015).

87. H. I. McKillop, *The Ancient Maya: New Perspectives* (Bloomsbury Publishing, 2004).

88. N. P. Dunning, T. Beach, "Farms and forests: Spatial and temporal perspectives on ancient Maya landscapes," in *Landscapes and Societies: Selected Cases,* P. Martini, W. Chesworth, Eds. (Springer, 2010), pp. 369–389.

89. K. M. Prufer, D. J. Kennett, "The Holocene occupations of southern Belize," in *Approaches to Monumental Landscapes of the Ancient Maya.,* B. A. Houk, B. Arroyo, T. G. Powis, Eds. (University Press of Florida, 2020), pp. 16–38.

90. K. M. Prufer, "Communities, caves, and ritual specialists: A study of sacred space in the Maya Mountains of southern Belize", thesis, Southern Illinois University Carbondale, St. Louis, MO, UMI (2002).

91. J. E. Brady, K. M. Prufer, *In The Maw of the Earth Monster: Mesoamerican Ritual Cave Use* (University of Texas Press, 2005).

92. K. M. Prufer, A. E. Thompson, D. J. Kennett, Evaluating airborne LiDAR for detecting settlements and modified landscapes in disturbed tropical environments at Uxbenká, Belize. *J. Archaeol. Sci.* **57**, 1–13 (2015).

93. B. J. Culleton, K. M. Prufer, D. J. Kennett, A bayesian AMS 14C chronology of the Classic Maya center of Uxbenká, Belize. *J. Archaeol. Sci.* **39**, 1572–1586 (2012).

94. K. M. Prufer, A. Thompson, A. Wickert, D. Kennett, The development and disintegration of a classic Maya center and its climate context. *Prog. Phys. Geogr.* **47**, 205–226 (2022).
