# Supplementary material for: Nutritional adaptations to early maize cultivation: Earliest isotopic evidence of maize-based animal provisioning in the Neotropics
Source: Sci Adv. 2026 Jul 8;12(28):eaec3522. doi: 10.1126/sciadv.aec3522 (PMC13344295; doi:10.1126/sciadv.aec3522)
Supplement: Supplementary file 3 — Codes S1 to S3 [file sciadv.aec3522_codes_s1_to_s3.zip › aec3522_code_s2.html]

Nutritional Adaptations to Early Maize Cultivation: Earliest Isotopic Evidence of Maize-Based Animal Husbandry in the Neotropics


Code 

- Show All Code
- Hide All Code

# Nutritional Adaptations to Early Maize Cultivation: Earliest Isotopic Evidence of Maize-Based Animal Husbandry in the Neotropics

#### Nadia Neff

#### October 25, 2025

**This document contains the code and output for all data based
figures in the manuscript and supplemental information.**

> Authors

Nadia C. Neff,1,3\* Geraldine
Busquets-Vass,2,3,4 Erin E. Ray,1,3 Mark
Robinson,5 Amy E. Thompson,6 Jose Mes,7
Douglas J. Kennett,8 Seth D. Newsome,2,3 Keith M.
Prufer1,3

> Affiliations

1Department of Anthropology, University of New Mexico,
Albuquerque, New Mexico, USA.  
2Department of Biology, University of New Mexico,
Albuquerque, New Mexico, USA.  
3Center for Stable Isotopes, University of New Mexico,
Albuquerque, New Mexico, USA.  
4Centro de Investigación Científica y de Educación Superior
de Ensenada, Unidad Académica La Paz, Baja California Sur, MEX.  
5Department of Archaeology, University of Exeter, Exeter,
Devon, UK.  
6Department of Geography and the Environment, University of
Texas at Austin, Austin, Texas, USA.  
7Uchben’kaj Kin Ahaw Association, Santa Cruz, Toledo
District, Belize.  
8Department of Anthropology, University of California, Santa
Barbara, California, USA.

```
#### CLEAN EVERYTHING ###
rm(list=ls())
graphics.off() # close all;
gc() # Clear memmory (residuals of operations?)
```

```
library(tidyverse)
library(ggplot2)
library(readxl)
library(stringr)
library(rcartocolor)
library(ggstar)
setwd("~/Library/Mobile Documents/com~apple~CloudDocs/R work/CSIA Stuff/Lysine")
#setwd("")
```

# 1 Data

## 1.1 δ13C Data

```
xlsx <- "Neff et al_Data S1.xlsx"
Iso_dat <- read_excel(xlsx
                      , sheet = 1
                      , skip = 1
                      )%>%
  mutate(
    Time_Bin = factor(Time_Bin
                    , levels = c(
                      "6,000+"
                    , "5,000-6,000"
                    , "4,000-5,000"
                    , "3,000-4,000"
                    , "2,000-3,000"
                    , "1,000-2,000"
                    )
                    )
    )
summary(Iso_dat)
```

```
  Burial_ID             Sex            Age_category           Age           
 Length:39          Length:39          Length:39          Length:39         
 Class :character   Class :character   Class :character   Class :character  
 Mode  :character   Mode  :character   Mode  :character   Mode  :character  
                                                                            
                                                                            
                                                                            
                                                                            
 Archaeological_Site        Time_Bin      14C_BP       14C_error    
 Length:39           6,000+     : 1   Min.   :1235   Min.   :15.00  
 Class :character    5,000-6,000: 5   1st Qu.:1512   1st Qu.:20.00  
 Mode  :character    4,000-5,000:10   Median :2270   Median :20.00  
                     3,000-4,000: 1   Mean   :2821   Mean   :22.69  
                     2,000-3,000: 3   3rd Qu.:4255   3rd Qu.:25.00  
                     1,000-2,000:19   Max.   :5300   Max.   :40.00  
                                                                    
   CalBP_Mean    CalBP_Median     Bulk13C          Bulk15N      
 Min.   :1109   Min.   :1107   Min.   :-22.00   Min.   : 6.300  
 1st Qu.:1370   1st Qu.:1370   1st Qu.:-20.50   1st Qu.: 7.250  
 Median :2025   Median :2025   Median :-11.10   Median : 7.900  
 Mean   :2923   Mean   :2920   Mean   :-14.28   Mean   : 8.074  
 3rd Qu.:4678   3rd Qu.:4679   3rd Qu.: -9.40   3rd Qu.: 8.600  
 Max.   :6093   Max.   :6093   Max.   : -8.20   Max.   :10.500  
                                                                
      C/N            Lys13C         Lys13C.SD          Leu13C      
 Min.   :2.700   Min.   :-23.56   Min.   :0.0000   Min.   :-30.00  
 1st Qu.:2.800   1st Qu.:-20.34   1st Qu.:0.0900   1st Qu.:-28.93  
 Median :3.200   Median :-16.61   Median :0.1600   Median :-20.56  
 Mean   :3.132   Mean   :-17.27   Mean   :0.2145   Mean   :-23.27  
 3rd Qu.:3.315   3rd Qu.:-14.76   3rd Qu.:0.3200   3rd Qu.:-19.48  
 Max.   :3.470   Max.   :-10.23   Max.   :0.5100   Max.   :-16.08  
                                  NA's   :6                        
   Leu13C.SD          Ile13C         Ile13C.SD          Phe13C      
 Min.   :0.0100   Min.   :-25.16   Min.   :0.0000   Min.   :-28.80  
 1st Qu.:0.0900   1st Qu.:-20.95   1st Qu.:0.1000   1st Qu.:-25.35  
 Median :0.2000   Median :-13.17   Median :0.1900   Median :-19.80  
 Mean   :0.2306   Mean   :-15.82   Mean   :0.2627   Mean   :-20.95  
 3rd Qu.:0.3600   3rd Qu.:-11.69   3rd Qu.:0.4400   3rd Qu.:-17.00  
 Max.   :0.6000   Max.   : -8.86   Max.   :0.7500   Max.   :-13.92  
 NA's   :6                         NA's   :6                        
   Phe13C.SD          Val13C         Val13C.SD      14C and Bulk Data source
 Min.   :0.0200   Min.   :-29.64   Min.   :0.0000   Length:39               
 1st Qu.:0.1800   1st Qu.:-26.00   1st Qu.:0.0800   Class :character        
 Median :0.2600   Median :-18.47   Median :0.1300   Mode  :character        
 Mean   :0.2821   Mean   :-20.17   Mean   :0.1952                           
 3rd Qu.:0.3900   3rd Qu.:-15.19   3rd Qu.:0.2500                           
 Max.   :0.5900   Max.   :-10.64   Max.   :0.6700                           
 NA's   :6                         NA's   :6
```

```
Iso_long <- 
  Iso_dat%>%
  select(
    Burial_ID
  , Time_Bin  
  , CalBP_Median
  , Lys13C
  , Val13C
  , Leu13C
  , Ile13C
  , Phe13C
  )%>%
  pivot_longer(
    cols = c(
    "Lys13C"
  , "Val13C"
  , "Leu13C"
  , "Ile13C"
  , "Phe13C"
  )
  , names_to = "AA"
  , values_to = "d13C"
  )%>%
  mutate(
    AA = recode_factor(AA
                      , Lys13C = "Lysine"
                      , Val13C = "Valine"
                      , Leu13C = "Leucine"
                      , Ile13C = "Isoleucine"
                      , Phe13C = "Phenylalanine"
                               )
  )
```

## 1.2 Model Data

```
xlsx  <- "Neff et al_Data S1.xlsx"
sheet <- 2

#Read the two header rows we want to combine (rows 3–4)
hdr <- read_excel(
    xlsx
  , sheet = sheet
  , range = cell_rows(3:4)
  , col_names = FALSE
)

#forward-fill NAs left-to-right for merged header cells from row 3
fill_forward <- function(x) {
  for (i in seq_along(x)) if (is.na(x[i]) && i > 1) x[i] <- x[i - 1]
  x
}

row3 <- as.vector(unlist(hdr[1, ], use.names = FALSE)) |> fill_forward()
row4 <- as.vector(unlist(hdr[2, ], use.names = FALSE))

#Combine row3 and row4 into clean column names like 'Lys_Mean', 'Lys_SD', etc.
raw_names <- mapply(
  function(a, b) {
    a <- ifelse(is.na(a), "", a)
    b <- ifelse(is.na(b), "", b)
    nm <- paste(a, b, sep = "_")
    nm <- gsub("^_|_$", "", nm)  # trim leading/trailing underscores
    nm
  },
  row3, row4, USE.NAMES = FALSE
)

# Clean to syntactically safe, compact snake-ish names
col_names <- raw_names |>
  str_replace_all("[^A-Za-z0-9]+", "_") |>
  str_replace_all("^_|_$", "") |>
  make.names(unique = TRUE)

#Read the actual data starting at row 5 using the new names
Model_dat <- read_excel(
    xlsx
  , sheet = sheet
  , skip = 4                   # skip rows 1–4 so data starts at row 5
  , col_names = col_names
)
summary(Model_dat)
```

```
  Burial_ID           Time_Bin          CalBP_Median  All_Essential_Model_Mean
 Length:39          Length:39          Min.   :1107   Min.   :0.1070          
 Class :character   Class :character   1st Qu.:1370   1st Qu.:0.2130          
 Mode  :character   Mode  :character   Median :2025   Median :0.7600          
                                       Mean   :2920   Mean   :0.5401          
                                       3rd Qu.:4679   3rd Qu.:0.8080          
                                       Max.   :6093   Max.   :0.8730          
                                                                              
 All_Essential_Model_SD Lys_model_Mean    Lys_model_SD     Val_model_Mean  
 Min.   :0.0390         Min.   :0.1410   Min.   :0.07500   Min.   :0.0480  
 1st Qu.:0.0440         1st Qu.:0.1975   1st Qu.:0.07800   1st Qu.:0.1065  
 Median :0.0520         Median :0.3690   Median :0.09300   Median :0.4770  
 Mean   :0.0529         Mean   :0.3537   Mean   :0.09477   Mean   :0.3398  
 3rd Qu.:0.0575         3rd Qu.:0.4810   3rd Qu.:0.10400   3rd Qu.:0.5215  
 Max.   :0.0860         Max.   :0.5960   Max.   :0.15100   Max.   :0.5790  
                                                                           
  Val_model_SD     Leu_model_Mean    Leu_model_SD     Ile_model_Mean  
 Min.   :0.04500   Min.   :0.4610   Min.   :0.02000   Min.   :0.1570  
 1st Qu.:0.06350   1st Qu.:0.4840   1st Qu.:0.02950   1st Qu.:0.2830  
 Median :0.06600   Median :0.9690   Median :0.03800   Median :0.8830  
 Mean   :0.06903   Mean   :0.7488   Mean   :0.08464   Mean   :0.6296  
 3rd Qu.:0.07250   3rd Qu.:0.9750   3rd Qu.:0.14100   3rd Qu.:0.9060  
 Max.   :0.10800   Max.   :0.9820   Max.   :0.16400   Max.   :0.9280  
                                                                      
  Ile_model_SD    Phe_model_Mean   Phe_model_SD    
 Min.   :0.0460   Min.   :0.112   Min.   :0.07200  
 1st Qu.:0.0555   1st Qu.:0.167   1st Qu.:0.07600  
 Median :0.0710   Median :0.577   Median :0.09100  
 Mean   :0.1021   Mean   :0.459   Mean   :0.09536  
 3rd Qu.:0.1455   3rd Qu.:0.711   3rd Qu.:0.09550  
 Max.   :0.2140   Max.   :0.781   Max.   :0.19700  
                                                   
 Lys_Daily_C4_requirements_Total_daily_requirement
 Min.   : 783.7                                   
 1st Qu.: 816.3                                   
 Median : 816.3                                   
 Mean   : 835.5                                   
 3rd Qu.: 816.3                                   
 Max.   :1696.1                                   
                                                  
 Lys_Daily_C4_requirements_Mean_C4 Lys_Daily_C4_requirements_SD_C4
 Min.   :115.1                     Min.   : 8.926                 
 1st Qu.:161.2                     1st Qu.:12.740                 
 Median :313.5                     Median :34.315                 
 Mean   :292.4                     Mean   :28.904                 
 3rd Qu.:392.7                     3rd Qu.:38.488                 
 Max.   :486.5                     Max.   :58.384                 
                                                                  
 Val_Daily_C4_requirements_Total_daily_requirement
 Min.   :465.3                                    
 1st Qu.:680.3                                    
 Median :680.3                                    
 Mean   :665.5                                    
 3rd Qu.:680.3                                    
 Max.   :963.7                                    
                                                  
 Val_Daily_C4_requirements_Mean_C4 Val_Daily_C4_requirements_SD_C4
 Min.   : 32.65                    Min.   : 1.469                 
 1st Qu.: 69.05                    1st Qu.: 4.385                 
 Median :310.88                    Median :22.241                 
 Mean   :226.44                    Mean   :16.563                 
 3rd Qu.:354.76                    3rd Qu.:24.090                 
 Max.   :393.88                    Max.   :40.629                 
                                                                  
 Leu_Daily_C4_requirements_Total_daily_requirement
 Min.   : 893.9                                   
 1st Qu.: 952.4                                   
 Median : 952.4                                   
 Mean   : 963.5                                   
 3rd Qu.: 952.4                                   
 Max.   :1619.0                                   
                                                  
 Leu_Daily_C4_requirements_Mean_C4 Leu_Daily_C4_requirements_SD_C4
 Min.   :435.3                     Min.   : 18.70                 
 1st Qu.:458.1                     1st Qu.: 26.96                 
 Median :870.6                     Median : 35.07                 
 Mean   :717.5                     Mean   : 48.53                 
 3rd Qu.:928.6                     3rd Qu.: 65.11                 
 Max.   :935.2                     Max.   :110.67                 
                                                                  
 Ile_Daily_C4_requirements_Total_daily_requirement
 Min.   : 379.6                                   
 1st Qu.: 680.3                                   
 Median : 680.3                                   
 Mean   : 659.7                                   
 3rd Qu.: 680.3                                   
 Max.   :1079.4                                   
                                                  
 Ile_Daily_C4_requirements_Mean_C4 Ile_Daily_C4_requirements_SD_C4
 Min.   : 72.88                    Min.   : 11.22                 
 1st Qu.:174.83                    1st Qu.: 25.43                 
 Median :534.69                    Median : 31.68                 
 Mean   :417.16                    Mean   : 34.44                 
 3rd Qu.:616.33                    3rd Qu.: 36.06                 
 Max.   :631.29                    Max.   :113.84                 
                                                                  
 Phe_Daily_C4_requirements_Total_daily_requirement
 Min.   :844.9                                    
 1st Qu.:952.4                                    
 Median :952.4                                    
 Mean   :938.7                                    
 3rd Qu.:952.4                                    
 Max.   :952.4                                    
                                                  
 Phe_Daily_C4_requirements_Mean_C4 Phe_Daily_C4_requirements_SD_C4
 Min.   :106.7                     Min.   : 9.28                  
 1st Qu.:151.4                     1st Qu.:14.08                  
 Median :549.5                     Median :48.67                  
 Mean   :432.7                     Mean   :39.40                  
 3rd Qu.:670.5                     3rd Qu.:52.52                  
 Max.   :743.8                     Max.   :85.55                  
                                                                  
 Lys_daily_maize_g_Mean Lys_daily_maize_g_SD Val_daily_maize_g_Mean
 Min.   : 852.6         Min.   : 66.12       Min.   : 10.08        
 1st Qu.:1194.3         1st Qu.: 94.37       1st Qu.: 21.31        
 Median :2322.0         Median :254.19       Median : 95.95        
 Mean   :2165.7         Mean   :214.10       Mean   : 69.89        
 3rd Qu.:2908.5         3rd Qu.:285.10       3rd Qu.:109.49        
 Max.   :3603.9         Max.   :432.47       Max.   :121.57        
                                                                   
 Val_daily_maize_g_SD Leu_daily_maize_g_Mean Leu_daily_maize_g_SD
 Min.   : 0.4535      Min.   :25.87          Min.   :1.111       
 1st Qu.: 1.3533      1st Qu.:27.22          1st Qu.:1.602       
 Median : 6.8647      Median :51.73          Median :2.084       
 Mean   : 5.1119      Mean   :42.63          Mean   :2.883       
 3rd Qu.: 7.4353      3rd Qu.:55.17          3rd Qu.:3.869       
 Max.   :12.5397      Max.   :55.57          Max.   :6.576       
                                                                 
 Ile_daily_maize_g_Mean Ile_daily_maize_g_SD Phe_daily_maize_g_Mean
 Min.   : 21.31         Min.   : 3.282       Min.   : 22.79        
 1st Qu.: 51.12         1st Qu.: 7.434       1st Qu.: 32.36        
 Median :156.34         Median : 9.262       Median :117.42        
 Mean   :121.98         Mean   :10.071       Mean   : 92.45        
 3rd Qu.:180.21         3rd Qu.:10.545       3rd Qu.:143.26        
 Max.   :184.59         Max.   :33.287       Max.   :158.93        
                                                                   
 Phe_daily_maize_g_SD Lys_daily_nix_g_Mean Lys_daily_nix_g_SD
 Min.   : 1.983       Min.   : 456.9       Min.   :243.0     
 1st Qu.: 3.009       1st Qu.: 638.0       1st Qu.:256.0     
 Median :10.399       Median :1195.6       Median :304.6     
 Mean   : 8.418       Mean   :1165.0       Mean   :313.0     
 3rd Qu.:11.223       3rd Qu.:1613.6       3rd Qu.:349.9     
 Max.   :18.281       Max.   :1931.1       Max.   :489.3     
                                                             
 Low_40_C4_derived_lysine_turkey_g_Mean Low_40_C4_derived_lysine_turkey_g_SD
 Min.   :13.32                          Min.   : 7.088                      
 1st Qu.:16.07                          1st Qu.: 7.277                      
 Median :19.28                          Median : 8.033                      
 Mean   :24.85                          Mean   : 8.667                      
 3rd Qu.:29.89                          3rd Qu.: 9.805                      
 Max.   :54.77                          Max.   :11.907                      
 NA's   :15                             NA's   :15                          
 Low_60_C4_derived_lysine_turkey_g_Mean Low_60_C4_derived_lysine_turkey_g_SD
 Min.   : 8.883                         Min.   :4.725                       
 1st Qu.:10.710                         1st Qu.:4.851                       
 Median :12.852                         Median :5.355                       
 Mean   :16.564                         Mean   :5.778                       
 3rd Qu.:19.926                         3rd Qu.:6.536                       
 Max.   :36.513                         Max.   :7.938                       
 NA's   :15                             NA's   :15                          
 High_C4_fauna_Mean High_C4_fauna_SD High_C4_plants_Mean High_C4_plants_SD
 Min.   :0.5920     Min.   :0.1170   Min.   :0.1430      Min.   :0.1170   
 1st Qu.:0.6870     1st Qu.:0.1310   1st Qu.:0.1470      1st Qu.:0.1310   
 Median :0.7740     Median :0.1480   Median :0.2260      Median :0.1480   
 Mean   :0.7565     Mean   :0.1587   Mean   :0.2435      Mean   :0.1587   
 3rd Qu.:0.8530     3rd Qu.:0.1825   3rd Qu.:0.3130      3rd Qu.:0.1825   
 Max.   :0.8570     Max.   :0.2190   Max.   :0.4080      Max.   :0.2190   
 NA's   :24         NA's   :24       NA's   :24          NA's   :24       
 High_C4_fauna_mg_Mean High_C4_fauna_mg_SD High_C4_plants_mg_Mean
 Min.   :227.7         Min.   : 4.143      Min.   : 54.80        
 1st Qu.:307.5         1st Qu.: 4.790      1st Qu.: 56.98        
 Median :328.7         Median : 6.106      Median : 96.12        
 Mean   :314.0         Mean   : 7.159      Mean   :105.73        
 3rd Qu.:330.3         3rd Qu.: 8.837      3rd Qu.:142.94        
 Max.   :335.6         Max.   :12.786      Max.   :198.50        
 NA's   :24            NA's   :24          NA's   :24            
 High_C4_plants_mg_SD High_40_C4_derived_lysine_turkey_g_Mean
 Min.   : 4.143       Min.   :26.35                          
 1st Qu.: 4.790       1st Qu.:35.59                          
 Median : 6.106       Median :38.04                          
 Mean   : 7.159       Mean   :36.35                          
 3rd Qu.: 8.837       3rd Qu.:38.23                          
 Max.   :12.786       Max.   :38.85                          
 NA's   :24           NA's   :24                             
 High_40_C4_derived_lysine_turkey_g_SD High_60_C4_derived_lysine_turkey_g_Mean
 Min.   :0.4795                        Min.   :17.57                          
 1st Qu.:0.5544                        1st Qu.:23.73                          
 Median :0.7067                        Median :25.36                          
 Mean   :0.8286                        Mean   :24.23                          
 3rd Qu.:1.0229                        3rd Qu.:25.48                          
 Max.   :1.4799                        Max.   :25.90                          
 NA's   :24                            NA's   :24                             
 High_60_C4_derived_lysine_turkey_g_SD High_Dry_maize_g_Mean
 Min.   :0.3197                        Min.   : 405.9       
 1st Qu.:0.3696                        1st Qu.: 422.1       
 Median :0.4711                        Median : 712.0       
 Mean   :0.5524                        Mean   : 783.2       
 3rd Qu.:0.6819                        3rd Qu.:1058.8       
 Max.   :0.9866                        Max.   :1470.4       
 NA's   :24                            NA's   :24           
 High_Dry_maize_g_SD High_Dry_nixtamalized_maize_g_Mean
 Min.   :30.69       Min.   :217.4                     
 1st Qu.:35.48       1st Qu.:226.1                     
 Median :45.23       Median :381.4                     
 Mean   :53.03       Mean   :419.6                     
 3rd Qu.:65.46       3rd Qu.:567.2                     
 Max.   :94.71       Max.   :787.7                     
 NA's   :24          NA's   :24                        
 High_Dry_nixtamalized_maize_g_SD
 Min.   :16.44                   
 1st Qu.:19.01                   
 Median :24.23                   
 Mean   :28.41                   
 3rd Qu.:35.07                   
 Max.   :50.74                   
 NA's   :24
```

# 2 Fig. 2: δ13Clysine Values of Individuals

```
library(rcartocolor)
p <- ggplot(Iso_dat
            , aes(x = reorder(Burial_ID, Lys13C)
                  , y = Lys13C
                  )
            )
p <- p + annotate("rect"
                  , xmin = -Inf
                  , xmax = Inf
                  , ymin = -15.91
                  , ymax = -15.84
                  , fill = "#CC79A7" 
                   )
p <- p + annotate("rect"
                  , xmin = -Inf
                  , xmax = Inf
                  , ymin = -9.11
                  , ymax = -9.06
                  , fill = "#CC79A7" 
                   )
p <- p + annotate("rect"
                  , xmin = -Inf
                  , xmax = Inf
                  , ymin = -21.51
                  , ymax = -21.46
                  , fill = "#333333" 
                   )

p <- p + annotate("rect"
                  , xmin = -Inf
                  , xmax = Inf
                  , ymin = -22.60
                  , ymax = -22.55
                  , fill = "#333333" 
                   )
p <- p + scale_color_carto_c("Cal BP"
                             , palette = "ag_Sunset"
                             )
p <- p + geom_point(aes(
  color = CalBP_Median
                      )
                    , size = 7
                    )
p <- p + scale_y_continuous(sec.axis = sec_axis(~.*1
                                                ))
p <- p + labs(
            title = "Lysine"
          , y = expression(paste(delta^{13}, "C (\u2030)"))
          , x = "Burial ID"
          , caption = ""
          )
p <- p + theme_light()
p <- p + theme(plot.caption = element_text(hjust = 0.5, face = "italic"))
p <- p + theme(plot.title = element_text(
                                           hjust = 0.5
                                         , face = "bold"
                                         , size = 30
                                         , family = "Times New Roman"
                                         )
               )
p <- p + theme(axis.title.y = element_text(
                                              family = "Times New Roman"
                                            , size   = 25
                                            , vjust  = 0.5
                                           )
               )
p <- p + theme(axis.text.y = element_text(
                                           face   = "bold"
                                         , size   = 20 
                                         , family = "Times New Roman"
                                         )
               )
p <- p + theme(axis.text.x = element_text(
                                           size   = 14    
                                         , family = "Times New Roman"
                                         , angle  = 90
                                         , hjust = 1
                                         , vjust = 0.5
                                         )
               )
p <- p + theme(axis.title.x = element_text(
                                          size   = 25    
                                        , family = "Times New Roman"
                                        , vjust  = 0.9
                                         )
               )
p <- p + theme(legend.text  = element_text(
                                              size   = 20
                                            , family = "Times New Roman"
                                            , face   = "bold"
                                            )
               )
p <- p + theme(legend.title  = element_blank())
p <- p + theme(panel.grid.major = element_blank()
               , panel.grid.minor = element_blank()
               )
p <- p + theme(legend.position = c(0.3, 0.8))
p <- p + theme(legend.background = element_rect(
                                    size     = 0.2
                                  , linetype = "solid"
                                  , colour   = "white"
                                  )
               )
p <- p + theme(
  strip.background = element_rect(
    color = "white"
  , fill = "white"
  )
)
p <- p + theme(strip.text = element_text(
                                             size   = 14
                                           , family = "Times New Roman"
                                           , face = "bold"
                                           , vjust  = 0.5
                                           , hjust  = 0
                                           , angle  = 0 
                                           )
               )
p <- p + theme(strip.text = element_text(
        size = 12
        , color = "black"
        )
      )
print(p)
```

# 3 Fig. 3: Lysine-based dietary calculations and results across time

## 3.1 δ13Clysine

```
p <- ggplot(Iso_dat
            , aes(x = reorder(Burial_ID, -CalBP_Median)
                  , y = Lys13C
                  )
            )
p <- p + scale_color_carto_c( "Median Cal. BP"
                             , palette = "ag_Sunset"
                             )
p <- p + geom_point(aes(color = CalBP_Median)
                    , size = 6
                    , alpha = 0.8 
                    )

p <- p + labs(
            title = ""
          , y = expression(paste(delta^{13}, "C Lysine (\u2030)"))
          , x = "Burial ID"
          , caption = ""
          )
p <- p + theme_light()
p <- p + theme(plot.caption = element_text(hjust = 0.5, face = "italic"))
p <- p + theme(plot.title = element_text(
                                           hjust = 0.5
                                         , face = "bold"
                                         , size = 30
                                         , family = "Times New Roman"
                                         )
               )
p <- p + theme(axis.title.y = element_text(
                                              family = "Times New Roman"
                                            , size   = 20
                                            , vjust  = 0.5
                                           )
               )
p <- p + theme(axis.text.y = element_text(
                                           face   = "bold"
                                         , size   = 15 
                                         , family = "Times New Roman"
                                         )
               )
p <- p + theme(axis.text.x = element_text(
                                           size   = 10    
                                         , family = "Times New Roman"
                                         , angle  = 90
                                         , hjust = 1
                                         , vjust = 0.5
                                         )
               )
p <- p + theme(axis.title.x = element_text(
                                          size   = 20    
                                        , family = "Times New Roman"
                                        , vjust  = 0.9
                                         )
               )
p <- p + theme(legend.text  = element_text(
                                              size   = 12
                                            , family = "Times New Roman"
                                            )
               )
p <- p + theme(legend.title  = element_text(
                                              size   = 14
                                            , family = "Times New Roman"
                                            , face   = "bold"
                                            )
               )
p <- p + theme(panel.grid.major = element_blank()
               , panel.grid.minor = element_blank()
               )
p <- p + theme(legend.position = "none")
p <- p + theme(legend.background = element_rect(
                                    size     = 0.2
                                  , linetype = "solid"
                                  , colour   = "white"
                                  )
               )
p <- p + facet_grid(cols = vars(Time_Bin)
                    , scales = "free_x"
                    , space = "free_x"
                    )
p <- p + theme(
  strip.background = element_rect(
    color = "white"
  , fill = "white"
  )
)
p <- p + theme(strip.text = element_text(
                                             size   = 10
                                           , family = "Times New Roman"
                                           , vjust  = 0.5
                                           , hjust  = 0
                                           , angle  = 90 
                                           )
               )
p <- p + theme(strip.text = element_text(
        size = 12
        , color = "black"
        )
      )
print(p)
```

## 3.2 Proportion of Lysine Derived from C4 Source

```
p <- ggplot(Model_dat
            , aes(x = reorder(Burial_ID, -CalBP_Median)
                  , y = Lys_model_Mean
                  )
            )

p <- p + scale_color_carto_c( "Median Cal. BP"
                             , palette = "ag_Sunset"
                             )
p <- p + geom_point(aes(color = CalBP_Median)
                    , size = 6
                    )
p <- p + geom_errorbar(aes(ymin = Lys_model_Mean-Lys_model_SD
                         , ymax =  Lys_model_Mean+Lys_model_SD
                         , color = CalBP_Median
                         )
                        , width = 0.2
                        , alpha = 0.5
                        , position = position_dodge(0.05)
                       )
p <- p + labs(
            title = ""
          , y = expression("Lysine Proportion C"[4])
          , x = "Burial ID"
          , caption = ""
          )

p <- p + theme_light()
p <- p + theme(plot.caption = element_text(hjust = 0.5, face = "italic"))
p <- p + theme(plot.title = element_text(
                                           hjust = 0.5 
                                         , face = "bold"
                                         , size = 30
                                         , family = "Times New Roman"
                                         )
               )
p <- p + theme(axis.title.y = element_text(
                                              family = "Times New Roman"
                                            , size   = 20
                                            , vjust  = 0.5
                                           )
               )
p <- p + theme(axis.text.y = element_text(
                                           face   = "bold"
                                         , size   = 15 
                                         , family = "Times New Roman"
                                         )
               )
p <- p + theme(axis.text.x = element_text(
                                           size   = 10    
                                         , family = "Times New Roman"
                                         , angle  = 90
                                         , hjust = 1
                                         , vjust = 0.5
                                         )
               )
p <- p + theme(axis.title.x = element_text(
                                          size   = 20    
                                        , family = "Times New Roman"
                                        , vjust  = 0.9
                                         )
               )
p <- p + theme(legend.text  = element_text(
                                              size   = 20
                                            , family = "Times New Roman"
                                            , face   = "bold"
                                            )
               )
p <- p + theme(legend.title  = element_blank())
p <- p + theme(panel.grid.major = element_blank()
               , panel.grid.minor = element_blank()
               )
p <- p + theme(legend.position = "none")
p <- p + theme(legend.background = element_rect(
                                    size     = 0.2
                                  , linetype = "solid"
                                  , colour   = "white"
                                  )
               )
p <- p + facet_grid(cols = vars(Time_Bin)
                    , scales = "free_x"
                    , space = "free_x"
                    )
p <- p + theme(
  strip.background = element_rect(
    color = "white"
  , fill = "white"
  )
)
p <- p + theme(strip.text = element_text(
                                             size   = 10
                                           , family = "Times New Roman"
                                           , vjust  = 0.5
                                           , hjust  = 0
                                           , angle  = 90 
                                           )
               )
p <- p + theme(strip.text = element_text(
        size = 12
        , color = "black"
        )
      )
print(p)
```

## 3.3 mg of Lysine Derived from C4 Source

```
p <- ggplot(Model_dat
            , aes(x = reorder(Burial_ID, -CalBP_Median)
                  , y = Lys_Daily_C4_requirements_Mean_C4
                  )
            )
p <- p + scale_color_carto_c( "Median Cal. BP"
                             , palette = "ag_Sunset"
                             )
p <- p + geom_point(aes(color = CalBP_Median)
                    , size = 6
                    )
p <- p + geom_errorbar(aes(ymin = Lys_Daily_C4_requirements_Mean_C4-Lys_Daily_C4_requirements_SD_C4
                         , ymax =  Lys_Daily_C4_requirements_Mean_C4+Lys_Daily_C4_requirements_SD_C4
                         , color = CalBP_Median
                         )
                        , width = 0.2
                        , alpha = 0.5
                        , position = position_dodge(0.05)
                       )

p <- p + labs(
            title = ""
          , y = expression("Daily mg C"[4] * " Lysine")
          , x = "Burial ID"
          , caption = ""
          )

p <- p + theme_light()
p <- p + theme(plot.caption = element_text(hjust = 0.5, face = "italic"))
p <- p + theme(plot.title = element_text(
                                           hjust = 0.5
                                         , face = "bold"
                                         , size = 30
                                         , family = "Times New Roman"
                                         )
               )
p <- p + theme(axis.title.y = element_text(
                                              family = "Times New Roman"
                                            , size   = 20
                                            , vjust  = 0.5
                                           )
               )
p <- p + theme(axis.text.y = element_text(
                                           face   = "bold"
                                         , size   = 15 
                                         , family = "Times New Roman"
                                         )
               )
p <- p + theme(axis.text.x = element_text(
                                           size   = 10    
                                         , family = "Times New Roman"
                                         , angle  = 90
                                         , hjust = 1
                                         , vjust = 0.5
                                         )
               )
p <- p + theme(axis.title.x = element_text(
                                          size   = 20    
                                        , family = "Times New Roman"
                                        , vjust  = 0.9
                                         )
               )
p <- p + theme(legend.text  = element_text(
                                              size   = 20
                                            , family = "Times New Roman"
                                            , face   = "bold"
                                            )
               )
p <- p + theme(legend.title  = element_blank())
p <- p + theme(panel.grid.major = element_blank()
               , panel.grid.minor = element_blank()
               )
p <- p + theme(legend.position = "none")
p <- p + theme(legend.background = element_rect(
                                    size     = 0.2
                                  , linetype = "solid"
                                  , colour   = "white"
                                  )
               )
p <- p + facet_grid(cols = vars(Time_Bin)
                    , scales = "free_x"
                    , space = "free_x"
                    )
p <- p + theme(
  strip.background = element_rect(
    color = "white"
  , fill = "white"
  )
)
p <- p + theme(strip.text = element_text(
                                             size   = 10
                                           , family = "Times New Roman"
                                           , vjust  = 0.5
                                           , hjust  = 0
                                           , angle  = 90 
                                           )
               )
p <- p + theme(strip.text = element_text(
        size = 12
        , color = "black"
        )
      )

print(p)
```

## 3.4 Grams of Maize Required to Meet Daily Lysine Needs

```
p <- ggplot(Model_dat
            , aes(x = reorder(Burial_ID, -CalBP_Median)
                  , y = Lys_daily_maize_g_Mean *3
                  )
            )
p <- p + annotate("rect"
                  , xmin = -Inf
                  , xmax = Inf
                  , ymin = 1400
                  , ymax = 2300
                  , fill = "#c2a4cb"
                  , alpha = 0.5
                   )
p <- p + scale_color_carto_c( "Median Cal. BP"
                             , palette = "ag_Sunset"
                             )
p <- p + geom_point(aes(color = CalBP_Median)
                    , size = 6
                    )
p <- p + geom_errorbar(aes(ymin = (Lys_daily_maize_g_Mean*3)-(Lys_daily_maize_g_SD *3)
                         , ymax =  (Lys_daily_maize_g_Mean*3)+(Lys_daily_maize_g_SD *3)
                         , color = CalBP_Median
                         )
                        , width = 0.2
                        , alpha = 0.5
                        , position = position_dodge(0.05)
                       )
p <- p + labs(
            title = ""
          , y = "Grams of Maize"
          , x = "Burial ID"
          , caption = ""
          )

p <- p + theme_light()
p <- p + theme(plot.caption = element_text(hjust = 0.5, face = "italic"))
p <- p + theme(plot.title = element_text(
                                           hjust = 0.5
                                         , face = "bold"
                                         , size = 30
                                         , family = "Times New Roman"
                                         )
               )
p <- p + theme(axis.title.y = element_text(
                                              family = "Times New Roman"
                                            , size   = 20
                                            , vjust  = 0.5
                                           )
               )
p <- p + theme(axis.text.y = element_text(
                                           face   = "bold"
                                         , size   = 15 
                                         , family = "Times New Roman"
                                         )
               )
p <- p + theme(axis.text.x = element_text(
                                           size   = 10    
                                         , family = "Times New Roman"
                                         , angle  = 90
                                         , hjust = 1
                                         , vjust = 0.5
                                         )
               )
p <- p + theme(axis.title.x = element_text(
                                          size   = 20    
                                        , family = "Times New Roman"
                                        , vjust  = 0.9
                                         )
               )
p <- p + theme(legend.text  = element_text(
                                              size   = 20
                                            , family = "Times New Roman"
                                            , face   = "bold"
                                            )
               )
p <- p + theme(legend.title  = element_blank())
p <- p + theme(panel.grid.major = element_blank()
               , panel.grid.minor = element_blank()
               )
p <- p + theme(legend.position = "none")
p <- p + theme(legend.background = element_rect(
                                    size     = 0.2
                                  , linetype = "solid"
                                  , colour   = "white"
                                  )
               )
p <- p + facet_grid(cols = vars(Time_Bin)
                    , scales = "free_x"
                    , space = "free_x"
                    )
p <- p + theme(
  strip.background = element_rect(
    color = "white"
  , fill = "white"
  )
)
p <- p + theme(strip.text = element_text(
                                             size   = 10
                                           , family = "Times New Roman"
                                           , vjust  = 0.5
                                           , hjust  = 0
                                           , angle  = 90
                                           )
               )
p <- p + theme(strip.text = element_text(
        size = 12
        , color = "black"
        )
      )
print(p)
```

# 4 Fig. 4: Maize (g) Required to Meet Daily Essential Amino Acid Requirements from C4 Source

```
EssAA_long_Mean <- Model_dat%>%
  select(
    Burial_ID
  , Time_Bin  
  , CalBP_Median
  , Lys_daily_maize_g_Mean
  , Val_daily_maize_g_Mean
  , Leu_daily_maize_g_Mean
  , Ile_daily_maize_g_Mean
  , Phe_daily_maize_g_Mean
  )%>%
  pivot_longer(cols = c("Lys_daily_maize_g_Mean"
                      , "Val_daily_maize_g_Mean"
                      , "Leu_daily_maize_g_Mean"
                      , "Ile_daily_maize_g_Mean"
                      , "Phe_daily_maize_g_Mean"
                      )
               , names_to = "AA.Mean"
               , values_to = "Means"
               )

EssAA_long_SD <- Model_dat%>%
  select(
    Lys_daily_maize_g_SD
  , Val_daily_maize_g_SD
  , Leu_daily_maize_g_SD
  , Ile_daily_maize_g_SD
  , Phe_daily_maize_g_SD
  )%>%
  pivot_longer(cols = c("Lys_daily_maize_g_SD"
                      , "Val_daily_maize_g_SD"
                      , "Leu_daily_maize_g_SD"
                      , "Ile_daily_maize_g_SD"
                      , "Phe_daily_maize_g_SD"
                      )
               , names_to = "AA.SD"
               , values_to = "SDs"
               )

EssAA_long <- cbind(EssAA_long_Mean, EssAA_long_SD)%>%
  select(
    Burial_ID
  , Time_Bin
  , CalBP_Median
  , AA.Mean
  , Means
  , AA.SD
  , SDs
  )%>%
  mutate(
    Time_Bin = factor(Time_Bin
                    , levels = c(
                      "6,000+"
                    , "5,000-6,000"
                    , "4,000-5,000"
                    , "3,000-4,000"
                    , "2,000-3,000"
                    , "1,000-2,000"
                    )
                    )
  , AA.Mean = recode_factor(AA.Mean
                            , Lys_daily_maize_g_Mean = "Lysine"
                            , Val_daily_maize_g_Mean = "Valine"
                            , Leu_daily_maize_g_Mean = "Leucine"
                            , Ile_daily_maize_g_Mean = "Isoleucine"
                            , Phe_daily_maize_g_Mean = "Phenylalanine"
                            )  
  )
```

```
library(ggstar)
p <- ggplot(EssAA_long
            , aes(x = reorder(Burial_ID, -CalBP_Median)
                  , y = Means *3 #a generalized conversion to maize wet weight to account for what people would have actually consumed
                  )
            )
p <- p + annotate("rect"
                  , xmin = -Inf
                  , xmax = Inf
                  , ymin = 1400
                  , ymax = 2300
                  , fill = "#c2a4cb"
                  , alpha = 0.5
                   )
p <- p + geom_errorbar(aes(ymin = (Means*3)-(SDs*3)
                         , ymax = (Means*3)+(SDs*3)
                         , color = CalBP_Median
                         )
                        , width = 0.2
                        , alpha = 0.5
                        , position = position_dodge(0.05)
                       )
p <- p + geom_star(aes(color = CalBP_Median
                    , fill =   CalBP_Median 
                    , starshape = AA.Mean)
                    , size = 6
                    , alpha = 0.8 
                    )
p <- p + scale_color_carto_c( "Median Cal. BP"
                             , palette = "ag_Sunset"
                             )
p <- p + scale_fill_carto_c( "Median Cal. BP"
                             , palette = "ag_Sunset"
                             )
p <- p + theme_light()
p <- p + labs(
            title = expression("Grams of Maize Derived from C"[4] * " Source to Meet Daily Needs")
          , y = "Grams"
          , x = "Burial ID"
          , caption = ""
          )
p <- p + theme(plot.caption = element_text(hjust = 0.5, face = "italic"))
p <- p + theme(plot.title = element_text(
                                           hjust = 0.5
                                         , face = "bold"
                                         , size = 24
                                         , family = "Times New Roman"
                                         )
               )
p <- p + theme(axis.title.y = element_text(
                                              family = "Times New Roman"
                                            , size   = 20
                                            , vjust  = 0.5
                                           )
               )
p <- p + theme(axis.text.y = element_text(
                                           face   = "bold"
                                         , size   = 15 
                                         , family = "Times New Roman"
                                         )
               )
p <- p + theme(axis.text.x = element_text(
                                           size   = 10    
                                         , family = "Times New Roman"
                                         , angle  = 90
                                         , hjust = 1
                                         , vjust = 0.5
                                         )
               )
p <- p + theme(axis.title.x = element_text(
                                          size   = 20    
                                        , family = "Times New Roman"
                                        , vjust  = 0.9
                                         )
               )
p <- p + theme(legend.text  = element_text(
                                              size   = 12
                                            , family = "Times New Roman"
                                            )
               )
p <- p + theme(legend.title  = element_text(
                                              size   = 14
                                            , family = "Times New Roman"
                                            , face   = "bold"
                                            )
               )
p <- p + theme(panel.grid.major = element_blank()
               , panel.grid.minor = element_blank()
               )
p <- p + theme(legend.position = "none")
p <- p + theme(legend.background = element_rect(
                                    size     = 0.2
                                  , linetype = "solid"
                                  , colour   = "white"
                                  )
               )
p <- p + facet_grid(cols = vars(Time_Bin)
                    , scales = "free_x"
                    , space = "free_x"
                    )
p <- p + theme(
  strip.background = element_rect(
    color = "white"
  , fill = "white"
  )
)
p <- p + theme(strip.text = element_text(
                                             size   = 14
                                           , family = "Times New Roman"
                                           , face = "bold"
                                           , vjust  = 0.5
                                           , hjust  = 0
                                           , angle  = 90
                                           )
               )
p <- p + theme(strip.text = element_text(
        size = 12
        , color = "black"
        )
      )
p <- p + theme(strip.text = element_text(angle = 0))
```

# 5 Fig. 5: Daily estimated quantities and combinations of food sources required

## 5.1 Fig 5A: Individuals with LOW δ13Clysine Values

```
LowLys <-  Model_dat%>%
  select(
    Burial_ID
  , Time_Bin  
  , CalBP_Median
  #, HighLow
  , Low_40_C4_derived_lysine_turkey_g_Mean
  , Lys_daily_maize_g_Mean
  , Lys_daily_nix_g_Mean
  )%>%
  filter(
    (!is.na(Low_40_C4_derived_lysine_turkey_g_Mean))
  )%>%
  pivot_longer(cols = c(
    #   "Mean.C4lbs.lys"
    # , "Lys_daily_nix_g_Mean"
     "Low_40_C4_derived_lysine_turkey_g_Mean"
    ,"Lys_daily_nix_g_Mean"
    , "Lys_daily_maize_g_Mean"
  ), names_to = "Type", values_to = "Mass")%>%
  mutate(
    Type = recode_factor(
      Type
      , Lys_daily_maize_g_Mean    = "Edible Maize Product"
      , Lys_daily_nix_g_Mean   = "Edible Nixtamalized Maize Product"
      , Low_40_C4_derived_lysine_turkey_g_Mean = "40% C4 Turkey Meat"
      )
  )
```

```
p <- ggplot(LowLys
            , aes(x = reorder(Burial_ID, -CalBP_Median)
                  , y = Mass * 3
                  , fill = Type
                  )
                  , colour = "#6a6a6a"
            )
p <- p + annotate("rect"
                  , xmin = -Inf
                  , xmax = Inf
                  , ymin = 1400
                  , ymax = 2300
                  , fill = "#c2a4cb"
                  , alpha = 0.5
                   )
p <- p + geom_bar(stat = "identity"
                  , position = position_dodge())
p <- p + ylim(0, 8400)
p <- p + labs(
            title = ""
          , y = "Grams"
          , x = "Burial ID"
          , caption = ""
          )
p <- p + scale_fill_manual(values =
                               c(
                                 "#c32c79"
                               , "#f89f77"
                               , "#7d2268"
                               , "#f05b6e"
                               , "#ffd9a0"
                               ))
p <- p + theme_light()
p <- p + theme(plot.caption = element_text(hjust = 0.5, face = "italic"))
p <- p + theme(plot.title = element_text(
                                           hjust = 0.5
                                         , face = "bold"
                                         , size = 30
                                         , family = "Times New Roman"
                                         )
               )
p <- p + theme(axis.title.y = element_text(
                                              family = "Times New Roman"
                                            , size   = 20
                                            , vjust  = 0.5
                                           )
               )
p <- p + theme(axis.text.y = element_text(
                                           face   = "bold"
                                         , size   = 15 
                                         , family = "Times New Roman"
                                         )
               )
p <- p + theme(axis.text.x = element_text(
                                           size   = 8    
                                         , family = "Times New Roman"
                                         , angle  = 90
                                         , hjust = 1
                                         , vjust = 0.5
                                         )
               )
p <- p + theme(axis.title.x = element_text(
                                          size   = 20    
                                        , family = "Times New Roman"
                                        , vjust  = 0.9
                                         )
               )
p <- p + theme(legend.text  = element_text(
                                              size   = 12
                                            , family = "Times New Roman"
                                            )
               )
               
p <- p + theme(legend.title  = element_blank())
p <- p + theme(panel.grid.major = element_blank()
               , panel.grid.minor = element_blank()
               )
p <- p + theme(legend.position = "bottom")
p <- p + theme(legend.background = element_rect(
                                    size     = 0.2
                                  , linetype = "solid"
                                  , colour   = "white"
                                  )
               )
p <- p + facet_grid(cols = vars(Time_Bin)
                    , scales = "free_x"
                    , space = "free_x"
                    )
p <- p + theme(
  strip.background = element_rect(
    color = "white"
  , fill = "white"
  )
)
p <- p + theme(strip.text = element_text(
                                             size   = 14
                                           , family = "Times New Roman"
                                           , face = "bold"
                                           , vjust  = 0.5
                                           , hjust  = 0
                                           , angle  = 90
                                           )
               )
p <- p + theme(strip.text = element_text(
        size = 12
        , color = "black"
        )
      )
print(p)
```

## 5.2 Fig. 5B: Individuals with HIGH δ13Clysine Values

```
HighLysmeans <-  Model_dat%>%
  select(
    Burial_ID
  , Time_Bin  
  , CalBP_Median
  , High_40_C4_derived_lysine_turkey_g_Mean
  , High_Dry_maize_g_Mean
  )%>%
  pivot_longer(cols = c(
     "High_40_C4_derived_lysine_turkey_g_Mean"
    , "High_Dry_maize_g_Mean"
  ), names_to = "Type", values_to = "Mass")

HighLysSD <- Model_dat%>%
  select(
    High_40_C4_derived_lysine_turkey_g_SD
  , High_Dry_maize_g_SD
  )%>%
  pivot_longer(cols = c(
     "High_40_C4_derived_lysine_turkey_g_SD"
    , "High_Dry_maize_g_SD"
    ), names_to = "AA.SD", values_to = "SDs")

HighLys <- cbind(HighLysmeans, HighLysSD)%>%
  na.omit()%>%
  mutate(
    Time_Bin = factor(Time_Bin)
  , Burial_ID = factor(Burial_ID)
  )%>%
  add_column(Diet = "A")
```

```
HighLysmeans <-  Model_dat%>%
  select(
    Burial_ID
  , Time_Bin  
  , CalBP_Median
  , High_60_C4_derived_lysine_turkey_g_Mean
  , High_Dry_nixtamalized_maize_g_Mean 
  )%>%
  pivot_longer(cols = c(
     "High_60_C4_derived_lysine_turkey_g_Mean"
    , "High_Dry_nixtamalized_maize_g_Mean"
  ), names_to = "Type", values_to = "Mass")

HighLysSD <- Model_dat%>%
  select(
    High_60_C4_derived_lysine_turkey_g_SD
  , High_Dry_nixtamalized_maize_g_SD
  )%>%
  pivot_longer(cols = c(
     "High_60_C4_derived_lysine_turkey_g_SD"
    , "High_Dry_nixtamalized_maize_g_SD"
    ), names_to = "AA.SD", values_to = "SDs")

HighLys2 <- cbind(HighLysmeans, HighLysSD)%>%
  na.omit()%>%
  mutate(
    Time_Bin = factor(Time_Bin)
  , Burial_ID = factor(Burial_ID))%>%
  add_column(Diet = "B")

HighLys <- rbind(HighLys, HighLys2)%>%
  mutate(
  Type = recode_factor(
    Type 
     , High_60_C4_derived_lysine_turkey_g_Mean = "60% C4 Turkey Meat"
     , High_Dry_nixtamalized_maize_g_Mean      = "Edible Nixtamalized Maize Product"
     , High_40_C4_derived_lysine_turkey_g_Mean = "40% C4 Turkey Meat"
     , High_Dry_maize_g_Mean                   = "Edible Maize Product"
  )
  )
```

```
p <- ggplot(HighLys
            , aes(x = Diet
                  , y = Mass * 3
                  , fill = Type
                  )
            ,  colour = "#3a3a3a"
            )
p <- p + annotate("rect"
                  , xmin = -Inf
                  , xmax = Inf
                  , ymin = 1400
                  , ymax = 2300
                  , fill = "#c2a4cb"
                  , alpha = 0.5
                   )
p <- p + geom_bar(
   stat = 'identity', position = 'stack')

p <- p + labs(
            title = ""
          , y = "Grams"
          , x = "Diet Combo"
          , caption = ""
          )
p <- p + scale_fill_manual(values =
                               c(
                                 "#f05b6e"
                               , "#f89f77"
                               , "#7d2268"
                               , "#c32c79"
                               , "#ffd9a0"
                               ))
p <- p + theme_light()
p <- p + theme(plot.caption = element_text(hjust = 0.5, face = "italic"))
p <- p + theme(plot.title = element_text(
                                           hjust = 0.5
                                         , face = "bold"
                                         , size = 30
                                         , family = "Times New Roman"
                                         )
               )
p <- p + theme(axis.title.y = element_text(
                                              family = "Times New Roman"
                                            , size   = 20
                                            , vjust  = 0.5
                                           )
               )
p <- p + theme(axis.text.y = element_text(
                                           face   = "bold"
                                         , size   = 15 
                                         , family = "Times New Roman"
                                         )
               )
p <- p + theme(axis.text.x = element_text(
                                           size   = 10    
                                         , family = "Times New Roman"
                                         , hjust = 1
                                         , vjust = 0.5
                                         )
               )
p <- p + theme(axis.title.x = element_text(
                                          size   = 20    
                                        , family = "Times New Roman"
                                        , vjust  = 0.9
                                         )
               )
p <- p + theme(legend.text  = element_text(
                                              size   = 12
                                            , family = "Times New Roman"
                                            )
               )
p <- p + theme(legend.title  = element_blank())
p <- p + theme(panel.grid.major = element_blank()
               , panel.grid.minor = element_blank()
               )
p <- p + theme(legend.position = "bottom")
p <- p + theme(legend.background = element_rect(
                                    size     = 0.2
                                  , linetype = "solid"
                                  , colour   = "white"
                                  )
               )
p <- p + facet_grid(~ Burial_ID
                    , scales = "fixed"
                    )
p <- p + theme(
  strip.background = element_rect(
    color = "white"
  , fill = "white"
  )
)
p <- p + theme(strip.text = element_text(
                                             size   = 8
                                           , family = "Times New Roman"
                                           , vjust  = 0.5
                                           , hjust  = 0
                                           , angle  = 90 
                                           , color  = "black" 
                                           )
               )
print(p)
```

# 6 Fig. S1: δ13C Essential Amino Acid Plots

```
p <- ggplot(Iso_long
            , aes(x = reorder(Burial_ID, -CalBP_Median)
                  , y = d13C
                  , group  = AA
                  )
            )
p <- p + scale_color_carto_c( "Median Cal. BP"
                             , palette = "ag_Sunset"
                             )
p <- p + geom_line(linetype = "dotted")
p <- p + geom_point(
                aes(color = CalBP_Median)
                    , size = 4
                    )
p <- p + labs(
            title = expression(paste(delta^{13}, "C (\u2030) Essential AAs"))
          , y = expression(paste(delta^{13}, "C (\u2030)"))
          , x = "Burial ID"
          , caption = ""
          )
p <- p + theme_light()
p <- p + theme(plot.caption = element_text(
                                            hjust = 0.5
                                          , face = "italic"
                                          )
               )
p <- p + theme(plot.title = element_text(
                                           hjust = 0.5 
                                         , face = "bold"
                                         , size = 30
                                         , family = "Times New Roman"
                                         )
               )
p <- p + theme(axis.title.y = element_text(
                                              family = "Times New Roman"
                                            , size   = 25
                                            , vjust  = 0.5
                                           )
               )
p <- p + theme(axis.text.y = element_text(
                                           face   = "bold"
                                         , size   = 15 
                                         , family = "Times New Roman"
                                         )
               )
p <- p + theme(axis.text.x = element_text(
                                           size   = 10    
                                         , family = "Times New Roman"
                                         , angle  = 90
                                         , hjust = 1
                                         , vjust = 0.5
                                         )
               )
p <- p + theme(axis.title.x = element_text(
                                          size   = 20    
                                        , family = "Times New Roman"
                                        , vjust  = 0.9
                                         )
               )
p <- p + theme(legend.text  = element_text(
                                              size   = 12
                                            , family = "Times New Roman"
                                            )
               )
p <- p + theme(legend.title  = element_text(
                                              size   = 14
                                            , family = "Times New Roman"
                                            , face   = "bold"
                                            )
               )
p <- p + theme(
                 panel.grid.major = element_blank()
               , panel.grid.minor = element_blank()
               )
p <- p + theme(legend.background = element_rect(
                                    size     = 0.2
                                  , linetype = "solid"
                                  , colour   = "white"
                                  )
               )
p <- p + facet_grid(AA ~. 
                  , scales = "free"  
                    )
p <- p + theme(
  strip.background = element_rect(
    color = "white"
  , fill = "white"
  )
)
p <- p + theme(strip.text = element_text(
                                             size   = 14
                                           , family = "Times New Roman"
                                           , face = "bold"
                                           , vjust  = 0.5
                                           , hjust  = 0
                                           , angle  = 90 
                                           )
               )
p <- p + theme(strip.text = element_text(
        size = 12
        , color = "black"
        )
      )
p <- p + theme(strip.text = element_text(angle = 0))
print(p)
```

# 7 Fig S2:Five Essential Amino Acids Mixing Model Results

```
p <- ggplot(Model_dat
            , aes(x = reorder(Burial_ID, -CalBP_Median)
                  , y = All_Essential_Model_Mean
                  )
            )
p <- p + scale_color_carto_c( "Median Cal. BP"
                             , palette = "ag_Sunset"
                             )
p <- p + geom_point(aes(
                    color = CalBP_Median
                    )
                    , size = 6
                    )
p <- p + geom_errorbar(aes(ymin = All_Essential_Model_Mean-All_Essential_Model_SD
                         , ymax = All_Essential_Model_Mean+All_Essential_Model_SD
                         , color = CalBP_Median
                         )
                       , width = 0.2
                       , alpha = 0.5
                       , position = position_dodge(0.05)
                       )
p <- p + labs(
            title = "All Essential AAs Model Results"
          , y = expression("Proportion C"[4])
          , x = "Burial ID"
          , caption = ""
          )
p <- p + theme_light()
p <- p + theme(plot.caption = element_text(hjust = 0.5, face = "italic"))
p <- p + theme(plot.title = element_text(
                                           hjust = 0.5 
                                         , face = "bold"
                                         , size = 30
                                         , family = "Times New Roman"
                                         )
               )
p <- p + theme(axis.title.y = element_text(
                                              family = "Times New Roman"
                                            , size   = 25
                                            , vjust  = 0.5
                                           )
               )
p <- p + theme(axis.text.y = element_text(
                                           face   = "bold"
                                         , size   = 20 
                                         , family = "Times New Roman"
                                         )
               )
p <- p + theme(axis.text.x = element_text(
                                           size   = 14    
                                         , family = "Times New Roman"
                                         , angle  = 90
                                         , hjust = 1
                                         , vjust = 0.5
                                         )
               )
p <- p + theme(axis.title.x = element_text(
                                          size   = 25    
                                        , family = "Times New Roman"
                                        , vjust  = 0.9
                                         )
               )
p <- p + theme(legend.text  = element_text(
                                              size   = 12
                                            , family = "Times New Roman"
                                            )
               )
p <- p + theme(legend.title  = element_text(
                                              size   = 14
                                            , family = "Times New Roman"
                                            , face   = "bold"
                                            )
               )
p <- p + theme(panel.grid.major = element_blank()
               , panel.grid.minor = element_blank()
               )
p <- p + theme(legend.background = element_rect(
                                    size     = 0.2
                                  , linetype = "solid"
                                  , colour   = "white"
                                  )
               )
p <- p + facet_grid(cols = vars(Time_Bin)
                    , scales = "free_x"
                    , space = "free_x"
                    )
p <- p + theme(
  strip.background = element_rect(
    color = "white"
  , fill = "white"
  )
)
p <- p + theme(strip.text = element_text(
                                             size   = 14
                                           , family = "Times New Roman"
                                           , face = "bold"
                                           , vjust  = 0.5
                                           , hjust  = 0
                                           , angle  = 90
                                           )
               )
p <- p + theme(strip.text = element_text(
        size = 12
        , color = "black"
        )
      )
p <- p + theme(strip.text = element_text(angle = 0))
print(p)
```

# 8 Fig. S3: Individual Essential Amino Acid Models Results

```
EssAA_long_Mean <- Model_dat%>%
  select(
    Burial_ID
  , Time_Bin  
  , CalBP_Median
  , Lys_model_Mean
  , Val_model_Mean
  , Leu_model_Mean
  , Ile_model_Mean
  , Phe_model_Mean
  )%>%
  pivot_longer(cols = c(
                        "Lys_model_Mean"
                      , "Val_model_Mean"
                      , "Leu_model_Mean"
                      , "Ile_model_Mean"
                      , "Phe_model_Mean"
                      )
               , names_to  = "AA.Mean"
               , values_to = "Means"
               )

EssAA_long_SD <- Model_dat%>%
  select(
    Lys_model_SD
  , Val_model_SD
  , Leu_model_SD
  , Ile_model_SD
  , Phe_model_SD
  )%>%
  pivot_longer(cols = c("Lys_model_SD"
                      , "Val_model_SD"
                      , "Leu_model_SD"
                      , "Ile_model_SD"
                      , "Phe_model_SD"
                      )
               , names_to  = "AA.SD"
               , values_to = "SDs"
               )

EssAA_long <- cbind(EssAA_long_Mean, EssAA_long_SD)%>%
  select(
    Burial_ID
  , Time_Bin 
  , CalBP_Median
  , AA.Mean
  , Means
  , AA.SD
  , SDs
  )%>%
  mutate(
    AA.Mean = recode_factor(AA.Mean
                              , Lys_model_Mean = "Lysine"
                              , Val_model_Mean = "Valine"
                              , Leu_model_Mean = "Leucine"
                              , Ile_model_Mean = "Isoleucine"
                              , Phe_model_Mean = "Phenylalanine"
                            )
  )
```

```
p <- ggplot(EssAA_long
            , aes(x = reorder(Burial_ID, -CalBP_Median)
                  , y = Means 
                  , group  = AA.Mean 
                  )
            )
p <- p + scale_color_carto_c( "Median Cal. BP"
                             , palette = "ag_Sunset"
                             )

p <- p + geom_errorbar(aes(ymin = Means-SDs
                         , ymax = Means+SDs
                         , color = CalBP_Median
                         )
                        , width = 0.2
                        , alpha = 0.5
                        , position = position_dodge(0.05)
                       )
p <- p + geom_point(aes(color = CalBP_Median)
                   , size = 4
                    )
p <- p + geom_line(linetype = "dotted")

p <- p + labs(
            title = "Individual Essential AA Model Results"
          , y = expression("Proportion C"[4])
          , x = "Burial ID"
          , caption = ""
          )
p <- p + theme_light()
p <- p + theme(plot.caption = element_text(hjust = 0.5, face = "italic"))
p <- p + theme(plot.title = element_text(
                                           hjust = 0.5
                                         , face = "bold"
                                         , size = 30
                                         , family = "Times New Roman"
                                         )
               )
p <- p + theme(axis.title.y = element_text(
                                              family = "Times New Roman"
                                            , size   = 25
                                            , vjust  = 0.5
                                           )
               )
p <- p + theme(axis.text.y = element_text(
                                           face   = "bold"
                                         , size   = 15 
                                         , family = "Times New Roman"
                                         )
               )
p <- p + theme(axis.text.x = element_text(
                                           size   = 10    
                                         , family = "Times New Roman"
                                         , angle  = 90
                                         , hjust = 1
                                         , vjust = 0.5
                                         )
               )
p <- p + theme(axis.title.x = element_text(
                                          size   = 20    
                                        , family = "Times New Roman"
                                        , vjust  = 0.9
                                         )
               )
p <- p + theme(legend.text  = element_text(
                                              size   = 12
                                            , family = "Times New Roman"
                                            )
               )
p <- p + theme(legend.title  = element_text(
                                              size   = 14
                                            , family = "Times New Roman"
                                            , face   = "bold"
                                            )
               )
p <- p + theme(panel.grid.major = element_blank()
               , panel.grid.minor = element_blank()
               )
p <- p + theme(legend.background = element_rect(
                                    size     = 0.2
                                  , linetype = "solid"
                                  , colour   = "white"
                                  )
               )
p <- p + facet_grid( AA.Mean ~ . 
                    , scales="fixed"
                                          )

p <- p + theme(
  strip.background = element_rect(
    color = "white"
  , fill = "white"
  )
)
p <- p + theme(strip.text = element_text(
                                             size   = 14
                                           , family = "Times New Roman"
                                           , face = "bold"
                                           , vjust  = 0.5
                                           , hjust  = 0
                                           , angle  = 90 
                                           )
               )
p <- p + theme(strip.text = element_text(
        size = 12
        , color = "black"
        )
      )
p <- p + theme(strip.text = element_text(angle = 0))
print(p)
```

# 9 Fig. S4: Daily mgs of Essential Amino Acids from a C4 Source

```
EssAA_long_Mean <- Model_dat%>%
  select(
    Burial_ID
  , Time_Bin  
  , CalBP_Median
  , Lys_Daily_C4_requirements_Mean_C4
  , Val_Daily_C4_requirements_Mean_C4
  , Leu_Daily_C4_requirements_Mean_C4
  , Ile_Daily_C4_requirements_Mean_C4
  , Phe_Daily_C4_requirements_Mean_C4
  )%>%
  pivot_longer(cols = c("Lys_Daily_C4_requirements_Mean_C4"
                      , "Val_Daily_C4_requirements_Mean_C4"
                      , "Leu_Daily_C4_requirements_Mean_C4"
                      , "Ile_Daily_C4_requirements_Mean_C4"
                      , "Phe_Daily_C4_requirements_Mean_C4"
                      )
               , names_to = "AA.Mean"
               , values_to = "Means"
               )

EssAA_long_SD <- Model_dat%>%
  select(
    Lys_Daily_C4_requirements_SD_C4
  , Val_Daily_C4_requirements_SD_C4
  , Leu_Daily_C4_requirements_SD_C4
  , Ile_Daily_C4_requirements_SD_C4
  , Phe_Daily_C4_requirements_SD_C4
  )%>%
  pivot_longer(cols = c("Lys_Daily_C4_requirements_SD_C4"
                      , "Val_Daily_C4_requirements_SD_C4"
                      , "Leu_Daily_C4_requirements_SD_C4"
                      , "Ile_Daily_C4_requirements_SD_C4"
                      , "Phe_Daily_C4_requirements_SD_C4"
                      )
               , names_to = "AA.SD"
               , values_to = "SDs"
               )

EssAA_long <- cbind(EssAA_long_Mean, EssAA_long_SD)%>%
  select(
    Burial_ID
  , Time_Bin
  , CalBP_Median
  , AA.Mean
  , Means
  , AA.SD
  , SDs
  )%>%
  mutate(
    AA.Mean = recode_factor(AA.Mean
                            , Lys_Daily_C4_requirements_Mean_C4 = "Lysine"
                            , Val_Daily_C4_requirements_Mean_C4 = "Valine"
                            , Leu_Daily_C4_requirements_Mean_C4 = "Leucine"
                            , Ile_Daily_C4_requirements_Mean_C4 = "Isoleucine"
                            , Phe_Daily_C4_requirements_Mean_C4 = "Phenylalanine"
                            )
  )
```

```
p <- ggplot(EssAA_long
            , aes(x = reorder(Burial_ID, -CalBP_Median)
                   , y = Means 
                   , group  = AA.Mean 
                  )
            )
p <- p + scale_color_carto_c( "Median Cal. BP"
                             , palette = "ag_Sunset"
                             )
p <- p + geom_line(linetype = "dotted")
p <- p + geom_errorbar(aes(ymin = Means-SDs
                         , ymax = Means+SDs
                         , color = CalBP_Median
                         )
                        , width = 0.2
                        , alpha = 0.5
                        , position = position_dodge(0.05)
                       )
p <- p + geom_point(aes(
                        color = CalBP_Median
                        )
                    , size = 4
                    )
p <- p + labs(
            title = expression("Daily mg of Essential AAs Derived from C"[4] * " Source")
          , y = "mg"
          , x = "Burial ID"
          , caption = ""
          )
p <- p + theme_light()
p <- p + theme(plot.caption = element_text(hjust = 0.5, face = "italic"))
p <- p + theme(plot.title = element_text(
                                           hjust = 0.5 
                                         , face = "bold"
                                         , size = 30
                                         , family = "Times New Roman"
                                         )
               )
p <- p + theme(axis.title.y = element_text(
                                              family = "Times New Roman"
                                            , size   = 25
                                            , vjust  = 0.5
                                           )
               )
p <- p + theme(axis.text.y = element_text(
                                           face   = "bold"
                                         , size   = 15 
                                         , family = "Times New Roman"
                                         )
               )
p <- p + theme(axis.text.x = element_text(
                                           size   = 10    
                                         , family = "Times New Roman"
                                         , angle  = 90
                                         , hjust = 1
                                         , vjust = 0.5
                                         )
               )
p <- p + theme(axis.title.x = element_text(
                                          size   = 20    
                                        , family = "Times New Roman"
                                        , vjust  = 0.9
                                         )
               )
p <- p + theme(legend.text  = element_text(
                                              size   = 12
                                            , family = "Times New Roman"
                                            )
               )
p <- p + theme(legend.title  = element_text(
                                              size   = 14
                                            , family = "Times New Roman"
                                            , face   = "bold"
                                            )
               )
p <- p + theme(panel.grid.major = element_blank()
               , panel.grid.minor = element_blank()
               )
p <- p + theme(legend.background = element_rect(
                                    size     = 0.2
                                  , linetype = "solid"
                                  , colour   = "white"
                                  )
               )
p <- p + facet_grid( AA.Mean ~ . 
                    , scales="fixed"
                                          )
p <- p + theme(
  strip.background = element_rect(
    color = "white"
  , fill = "white"
  )
)
p <- p + theme(strip.text = element_text(
                                             size   = 14
                                           , family = "Times New Roman"
                                           , face = "bold"
                                           , vjust  = 0.5
                                           , hjust  = 0
                                           , angle  = 90
                                           )
               )
p <- p + theme(strip.text = element_text(
        size = 12
        , color = "black"
        )
      )
p <- p + theme(strip.text = element_text(angle = 0))
print(p)
```
